# Nanoparticle Exsolution from Nanoporous Perovskites for Highly Active and Stable Catalysts

**DOI:** 10.1002/advs.202205890

**Published:** 2023-01-22

**Authors:** Benjamin Rudolph, Anastasios I. Tsiotsias, Benedikt Ehrhardt, Paolo Dolcet, Silvia Gross, Sylvio Haas, Nikolaos D. Charisou, Maria A. Goula, Simone Mascotto

**Affiliations:** ^1^ Institut für Anorganische und Angewandte Chemie Universität Hamburg Martin‐Luther‐King‐Platz, 6 20146 Hamburg Germany; ^2^ Department of Chemical Engineering University of Western Macedonia Koila Kozani 50100 Greece; ^3^ Institute for Chemical Technology and Polymer Chemistry Karlsruhe Institute of Technology Engesserstrasse 20 76133 Karlsruhe Germany; ^4^ Dipartimento di Scienze Chimiche Università degli Studi di Padova via Marzolo 1 Padova 35131 Italy; ^5^ Deutsches Elektronen Synchrotron (DESY) Notkestr. 85 22607 Hamburg Germany

**Keywords:** catalyst regeneration, CO_2_ conversion, hydrogen production, oxygen mobility, small‐angle X‐ray scattering

## Abstract

Nanoporosity is clearly beneficial for the performance of heterogeneous catalysts. Although exsolution is a modern method to design innovative catalysts, thus far it is predominantly studied for sintered matrices. A quantitative description of the exsolution of Ni nanoparticles from nanoporous perovskite oxides and their effective application in the biogas dry reforming is here presented. The exsolution process is studied between 500 and 900 °C in nanoporous and sintered La_0.52_Sr_0.28_Ti_0.94_Ni_0.06_O_3±*δ*_. Using temperature‐programmed reduction (TPR) and X‐ray absorption spectroscopy (XAS), it is shown that the faster and larger oxygen release in the nanoporous material is responsible for twice as high Ni reduction than in the sintered system. For the nanoporous material, the nanoparticle formation mechanism, studied by in situ TEM and small‐angle X‐ray scattering (SAXS), follows the classical nucleation theory, while on sintered systems also small endogenous nanoparticles form despite the low Ni concentration. Biogas dry reforming tests demonstrate that nanoporous exsolved catalysts are up to 18 times more active than sintered ones with 90% of CO_2_ conversion at 800 °C. Time‐on‐stream tests exhibit superior long‐term stability (only 3% activity loss in 8 h) and full regenerability (over three cycles) of the nanoporous exsolved materials in comparison to a commercial Ni/Al_2_O_3_ catalyst.

## Graphical abstract

The design of nanoporous oxides represents an appealing strategy for the development of exsolved materials. Herein, the process of metal exsolution from nanoporous perovskite oxides is thoroughly described and compared to the one from sintered matrices. The combination of support nanoporosity with the exsolution of metal nanoparticles leads to high‐performing, stable, and regenerable heterogeneous catalysts for biogas dry reforming.

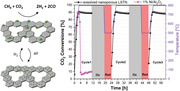

## 1 Introduction

The great challenge of our epoch is to drastically reduce greenhouse gas emissions, so as to mitigate their detrimental effect on global warming and our planet's climate.^[^ ^1^ ^]^ Coupled with energy insecurity, violently brought to the fore from recent global conflicts, the utilization of renewable and domestic energy resources is now more important than ever. Biogas originating from the fermentation of bio‐waste can be considered an attractive and renewable alternative power source.^[^ ^2^ ^]^ However, it first needs to undergo purification processes (e.g., drying, desulfurization), whereas its high CO_2_ content needs to be removed prior to the production of biomethane.^[^ ^3^ ^]^ Alternatively, biogas dry reforming has been demonstrated to be an efficient method to convert the two major components of biogas (CO_2_ and CH_4_) into useful synthesis gas, that is, an H_2_ and CO mixture, which can, in turn, be used for the production of value‐added chemicals and higher hydrocarbons.^[^ ^4^, ^5^ ^]^ Biogas dry reforming is similar to the methane dry reforming reaction (albeit with a higher CH_4_:CO_2_ ratio, which depends on the biogas composition), it is highly endothermic, and can be described by the following equation.^[^ ^4^, ^5^, ^6^ ^]^

(1) $$\;{\mathrm{CH}}_{4}+{\mathrm{CO}}_{2}\to 2H_{2}+2\mathrm{CO}$$

Besides noble metals, which are scarce and highly expensive, nickel‐based catalysts have been shown to be highly active for the CO_2_‐reforming of methane and biogas.^[^ ^7^, ^8^ ^]^ However, a major drawback of Ni‐based catalysts is their high tendency towards carbon accumulation, as carbon species originating from methane decomposition are incompletely removed from the metallic surface during the reaction, leading to the extensive build‐up of coke.^[^ ^9^, ^10^ ^]^ The encapsulation of Ni particles with carbon species reduces the catalytically active surface area, leading to severe catalyst degradation.^[^ ^10^, ^11^ ^]^ Great attempts have been undertaken to reduce the coking propensity of Ni catalysts, usually focusing on improving the Ni dispersion,^[^ ^9^ ^]^ the catalyst basicity,^[^ ^12^ ^]^ and the oxygen storage capacity.^[^ ^13^, ^14^ ^]^ One of the best strategies has been found to be the facilitation of strong‐metal support interactions induced via the in situ formation of Ni nanoparticles through the exsolution of Ni dopants previously incorporated into a metal oxide host.^[^ ^15^ ^]^ As such, Ni nanoparticles formed this way are strongly socketed in their support, they are much less prone to coking, and thereby typically exhibit superior stability under long‐term operation.^[^ ^16^, ^17^, ^18^ ^]^

The exsolution process proceeds with the reduction of a doped perovskite oxide when exposed to an oxygen‐poor atmosphere at high temperatures. Upon oxygen release from the lattice, transition metal dopants are reduced to the elemental state. After spontaneous nucleation and growth, metal nanoparticles emerge from the oxide structure, thus intrinsically forming supported metal catalysts strongly anchored in the parental matrix.^[^ ^19^ ^]^ An additional advantage with respect to impregnated systems is that the process of exsolution is cyclable.^[^ ^20^ ^]^ Upon high‐temperature oxidation, the metal nanoparticles dissolve back as dopants into the oxide matrix and after a second reduction treatment, they regenerate on the material surface.^[^ ^21^ ^]^ Hence, exsolution represents a smart, modern approach for the design of shape and size‐controlled sustainable, green catalysts.^[^ ^19^, ^22^ ^]^

So far, the exsolution process has been almost exclusively adopted for bulk, sintered matrices. Surprisingly, only very few works deal with metal exsolution from nanoporous and nanostructured perovskite oxides^[^ ^23^, ^24^ ^]^ and therefore there is very little knowledge on the exsolution process from these matrices. It is common knowledge that catalytic processes are favored on nanostructured and nanoporous materials due to higher specific surface area and higher concentration of active sites.^[^ ^25^, ^26^, ^27^ ^]^ Although highly promising, the combination of exsolved nanoparticles with nanoporous perovskite matrices has still to be demonstrated to be a valuable strategy for the design of highly active and stable heterogeneous catalysts.

Currently, it is completely unclear whether the attractive features of exsolved nanoparticles for high‐temperature catalysis, such as strong anchoring on the parental matrix and self‐regeneration, are also valid for nanoporous systems. Due to their high surface/volume ratio, nanoporous matrices are characterized by high surface energy and shorter diffusion path lengths of ions through the nanostructured grains.^[^ ^28^, ^29^, ^30^ ^]^ As we have previously shown, these features lead to a significant improvement in the mobility of oxygen anions^[^ ^31^, ^32^ ^]^ and metal dopants.^[^ ^33^, ^34^ ^]^ This enhanced ionic diffusion combined with the higher exposure to the hydrogen flow of the large surface area matrix, might favor the exsolution process by strongly decreasing the exsolution kinetics. Also, this modified, nanostructure‐driven, exsolution mechanism can possibly lead to exsolved nanoparticles with different features than the usually known sintered matrices.

This work provides a detailed description of the exsolution process of nickel from nanoporous and sintered La_0.52_Sr_0.28_Ti_0.94_Ni_0.06_O_3_ (LSTN) and their application as catalysts for the biogas dry reforming reaction. Due to their high thermal stability and a substantial concentration of cationic and oxygen defects, doped A‐site deficient SrTiO_3_‐based perovskite is among the most used matrices for exsolution and therefore represents a good model system for this study. The nanoporous and sintered samples were denoted as n‐LSTN and s‐LSTN, respectively, and exsolved systems as n‐ or s‐LSTNR*T* (with *T* the exsolution temperature in the range 500–900 °C). The use of a multi‐technique approach resulted fundamental to clearly understand the exsolution process of the Ni species. Thus, combining TPR with XAS, we identified the heterogeneous and large oxygen release as the real descriptor for the extensive Ni reduction in n‐LSTN. Also, SAXS showed to be an original and effective approach to shed new light into the nanoparticle growth mechanism in nanoporous and sintered systems, overcoming the instrumental limits of X‐ray diffraction (XRD) (crystallinity) and electron microscopy (statistical significance).

Biogas dry reforming was employed to test the performance of the nanoporous exsolved catalysts. The materials were far more active than the sintered counterparts at 800°C. The highly successful time‐on‐stream tests conducted over 60 h, demonstrated that nanoparticle stability and self‐regeneration are preserved in nanoporous materials, making these systems far superior to a commercial Ni/Al_2_O_3_ catalyst.

Thus, the design of nanoporous exsolved materials is an effective approach for the development of high‐performing heterogeneous catalysts. Also, the chance to control the reduction and growth process of exsolved metal nanoparticles by tuning the anionic and cationic mobility through the design of nanoporous parental structures with different porosity offers interesting prospects for the future design of advanced exsolved catalysts.

## 2 Results and Discussion

### 2.1 Parental Structures

**Figure 1:**
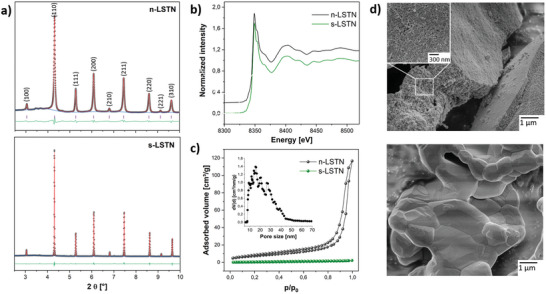
a) XRD patterns and b) X‐ray absorption spectra of the nanostructured (n‐LSTN) and sintered (s‐LSTN) parental structures; c) Nitrogen physisorption isotherms evidencing the nanoporous structure of n‐LSTN along with the pore size distribution (inset); d) SEM pictures of the nanostructured (top) and sintered (bottom) parental structures.

The crystalline nature of sintered and nanostructured perovskite parental structures has been investigated by XRD analyses (**Figure** **1**a). Both LSTN materials present a cubic Pm‐3m structure, as shown in similar works.^[^ ^35^ ^]^ The atomic ratios of the cations fit well with the nominal stoichiometry. While the sintered system shows oxygen deficiency (*δ* ≈ −0.2), n‐LSTN is characterized by a stoichiometric oxygen content (Tables S1, and S2, Supporting Information). The different oxygen stoichiometry in the nanoporous matrix is likely related to the highly defective surface and nanostructured grains (average crystallite size of 22 nm) as a larger concentration of defects (e.g., stacking faults, grain boundaries) would favor the oxygen incorporation during the annealing step. Also, the formation of Ruddlesden–Popper units presenting an additional SrO layer cannot be excluded.^[^ ^36^, ^37^, ^38^ ^]^ To clarify the state of the Ni dopant, XAS analyses were performed at the Ni K edge (*E*_Ni_ = 8333 eV) of both nanoporous and sintered materials. X‐ray absorption near edge structure (XANES) spectra (Figure 1b) are indicative of octahedral Ni (^oct^Ni^n+^) occupying Ti sites in a titanate perovskite structure, as evidenced by the shoulder on the high‐energy flank of its whiteline.^[^ ^39^, ^40^ ^]^ The edge position (*E*_LSTN_ = 8347 eV) suggests an average Ni oxidation state (*n*) of +2.5.^[^ ^40^, ^41^ ^]^ Extendedray X‐ray absorption fine structure (EXAFS) analyses confirmed for both cases an octahedral coordination around the absorbing Ni atom, with a Ni–O bond distance (≈ 2.01 ± 0.01 Å, **Table** **1**) in line with the LSTN structure. The second shell contribution (bond distance ≈ 2.95 Å) also corresponds to the Ni–M distance usually found in such perovskite structures (Table S3, Supporting Information).

**Table 1:** Lattice parameter and average crystallite size (*φ*) from the XRD data; specific surface area (SSA), pore volume (*V*_P_) from nitrogen physisorption; relative amount of metallic Ni from XANES analyses; Ni–Ni and Ni–O distances from EXAFS analyses (coordination numbers set to 12 and 6, respectively); Ni nanoparticle size obtained from electron microscopy (*D*_EM_) and SAXS (*2R*_SAXS_); normalized number density times scattering contrast square of particles from SAXS (N*η*2)

Sample	*φ* [nm]	SSA [m^2^/g]	V_P_ [cm^3^/g]	Ni^0^ [%]	Ni‐O [Å]	Ni‐Ni [Å]	D_EM_ [nm]	2R_SAXS_ [nm]	N*η* ^2^
s‐LSTN	–	1	–	–	2.02 (1)		–	–	–
s‐LSTNR700	–	1	–	7.0 ± 3.3	2.02 (1)	2.49 (1)	15	–	–
s‐LSTNR800	–	1	–	32.1 ± 0.2	2.01 (1)	2.51 (1)	22	–	–
s‐LSTNR900	–	1	–	38.5 ± 2.3	2.01 (1)	2.51 (1)	30	8	–
n‐LSTN	22	26	0.16	–	2.02 (1)		–	–	–
n‐LSTNR500	23	23	0.13	7.7 ± 1.3	2.02 (1)	2.49 (1)	12	14.2 (3)	1.00 (0)
n‐LSTNR700	23	24	0.14	32.0 ± 2.5	2.01 (1)	2.50 (1)	17	14.8 (5)	0.86 (9)
n‐LSTNR800	24	22	0.13	63.3 ± 0.3	2.01 (1)	2.49 (1)	20	17.0 (2)	0.66 (3)
n‐LSTNR900	33	19	0.12	74.1 ± 2.5	2.00 (1)	2.49 (2)	36	19.2 (3)	0.33 (7)

From nitrogen physisorption analyses, n‐LSTN displays a typical type IV isotherm distinctive of a nanoporous structure with an average pore size of 20 nm (Figure 1c). The material presents a specific surface area (SSA) of 26 m^2^ g^−1^, while the sintered counterpart of only 1 m^2^ g^−1^. In SEM pictures the nanoporous and sintered nature of the materials is clearly distinguishable (Figure 1d). Sintered LSTN displays grains of size varying between 1 to 3 µm, while n‐LSTN shows a nanoporous structure with pores of 20 nm in agreement with nitrogen physisorption analysis. Elemental analysis using energy dispersive X‐ray spectroscopy (EDX) from SEM is in good agreement with the theoretical values (Table S4 and Figure S1, Supporting Information). XPS analyses show only signals of the elements of interest, indicating the high purity level of the materials (Figure S2, Supporting Information). Due to the overlap between the Ni 2p and the La 3d_3/2_ spectra, no information regarding the status of Ni could be obtained.

### 2.2 Exsolved Materials

**Figure 2:**
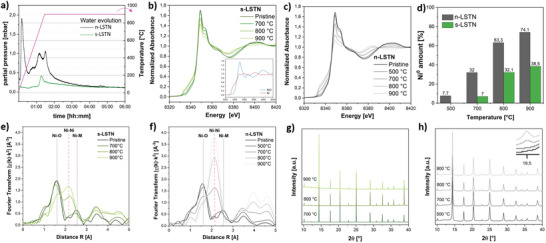
a) Water evolution during exsolution from mass spectrometry; b) XANES spectra of the sintered (reference spectra of Ni^0^ and NiO in the inset) and c) nanostructured LSTN after exsolution at different temperatures; c) Relative amounts of Ni^0^ in s‐LSTN and n‐LSTN resulted from the linear combination fitting of the XANES spectra at different exsolution temperatures; Fourier transforms of the *k* ^2^‐weighted EXAFS curves of the e) sintered and f) nanostructured LSTN; XRD patterns of g) s‐LSTN and h) n‐LSTN after exsolution at different temperatures. The inset in h) shows the evolution of the (111) reflection of the metallic Ni particles in n‐LSTN.

The process of exsolution was firstly monitored with mass spectrometry to get insights into the reduction of the materials during the high‐temperature treatment with hydrogen. Here the evolution of the water signal was followed (**Figure** **2**a) since it is directly correlated to the release of lattice oxygen and thus to the reduction of Ni^n+^:

(2a) $$H_{2}+O^{2-}\to H_{2}O+2e^{-}$$

(2b) $${\mathrm{Ni}}^{n+}+{\mathrm{ne}}^{-}\to {\mathrm{Ni}}^{0}$$

The nanoporous sample presents a much larger water release than the sintered one. In particular, besides an intense release of physisorbed water at T ≈ 100 °C due to its large SSA, n‐LSTN presents a significant water signal starting at ≈ 300 °C. This might be assigned to the release of surface oxygen species (*α*‐oxygen), which play an important role in the reactivity of mesoporous and nanostructured perovskite oxides.^[^ ^28^, ^31^, ^32^ ^]^ A further important release event at 700 °C is observed for both nanoporous and sintered systems and is likely correlated to the lattice oxygen (*β*‐oxygen) release. n‐LSTN displays a third signal at 900 °C, which cuts off when the temperature plateau sets in, and it might be caused by the release of lattice oxygen in a different configuration, which is not present in the sintered system.

The study of the state and speciation of nickel in the materials after reduction was performed using X‐ray absorption spectroscopy. Taking into account the low Ni concentration in the perovskite, the high sensitivity of this method enables precise assessment of the Ni reduction both at the surface and in the bulk.

From XANES spectra (Figure 2b,c), a gradual shift of the absorption edge to lower energy is observed upon increasing the reduction temperature and indicates a decrease in the oxidation state of the metal. Also, the emergence of the edge feature at ≈ 8335 eV and the lowering of the white line intensity are typical of the formation of metallic Ni species. Linear combination fitting (LCF) analyses based on the reference spectra of metallic Ni, NiO (inset in Figure 2b), and pristine LSTN was applied to quantify the contribution of Ni^0^, Ni^2+^, and ^oct^Ni^n+^ species, respectively, after reduction (Table S6 and Figure S3, Supporting Information). Clearly, none of the reduced materials presents the formation of NiO. As expected, in both nanostructured and sintered systems, the concentration of metallic Ni increases proportionally with the treatment temperature (Figure 2d). The extent of reduced Ni is found to be much larger in n‐LSTN than in s‐LSTN and reached 74.1% at 900 °C. While the nanoporous material displays 7.7% Ni^0^ already at 500 °C, the Ni reduction starts at 700 °C in the sintered system, in accordance with the minimum temperature required for the *β*‐oxygen release. From the Arrhenius plots of the extent of reduced nickel (Figure S4, Supporting Information), the activation energy of Ni reduction for the nanoporous system (45 ± 3 kJ∙mol^−1^) was almost half of the one of s‐LSTN (82 ± 32 kJ∙mol^‐1^). As oxygen release is the main driving force for exsolution, from these analyses it is clear that in nanoporous systems the exsolution process is favored for two reasons. Firstly, due to the larger SSA, the early and prominent *α*‐oxygen release initiates the reduction process at a temperature as low as 500 °C. Secondly, the more defective grain structure and shorter ion diffusion length pathways proper of the nanostructured material allow larger storage and a more prominent release of *β*‐oxygen^[^ ^31^ ^]^ through the A‐site deficient lattice, thus easing and maximizing the reduction of Ni dopants. Therefore, by controlling the nanoporous structure of the matrix major control over the reduction step of exsolution is achieved.

**Figure 3:**
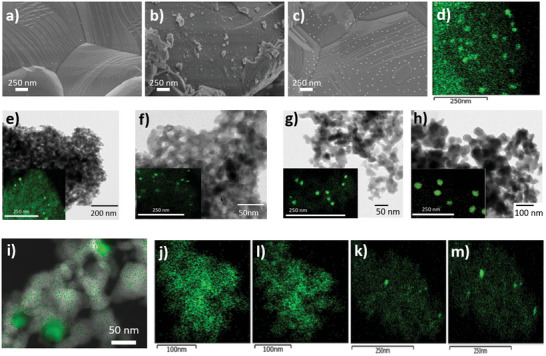
SEM images of s‐LSTN exsolved at a) 700, b) 800, and c) 900 °C; d) TEM‐EDX mapping of Ni of s‐LSTNR900; TEM images and respective Ni‐EDX mapping (inset) of n‐LSTN exsolved at e) 500, f) 700, g) 800, and h) 900 °C; i) superposition of STEM and respective Ni‐EDX mapping of n‐LSTNR900 indicating the Ni enrichment in the perovskite matrix in the proximity of the exsolved Ni nanoparticles; Ni‐EDX mapping from in situ TEM of n‐LSTN under vacuum at j) 400, l) 500, k) 700, and m) 800 °C, the acquisition spot was changed after the acquisition at 500 °C in order to avoid beam damage from overexposure.

The enhanced reducibility of the nanoporous samples is also confirmed by analysis of the EXAFS curves (Figure 2e,f, and Figures S5 and S6, Supporting Information). While for the sintered samples the contributions due to the metallic Ni (peak at ≈2.1 Å and signals in the range 3.5–5 Å in Figure 2e,f) are evident only for temperatures of at least 800 °C, in the nanoporous series these signals are already very intense at 700°C. A detailed fitting of these curves (Table S3, Supporting Information), further reveals that the trends in Ni° content (as Ni–Ni_metallic Ni_) are in good agreement with the values determined by LCF of the XANES region, especially for the higher temperatures. For example, a Ni° content (as Ni–Ni_metallic Ni_) of 11.8% is found already for the 500 °C sample, while for the 900 °C sample this content increases to 79% (74.1% from XANES LCF). It is thus clear that with increasing reduction temperature the average local structure around the Ni becomes increasingly metal‐like, as a consequence of the exsolution process. No significant changes in the Ni–O or Ni–Ni distances can be seen from the results of the fitting. Furthermore, in all samples, the metallic component presents a common Ni–Ni coordination number of 12, as the bulk metallic fcc Ni, and this reveals the formation of nanoparticles bigger than ≈5–6 nm for all the samples, as can be inferred from geometrical considerations.^[^ ^42^, ^43^ ^]^ Complementary to EXAFS, XRD analyses (Figure 2g,h) also clearly show the formation of Ni nanoparticles in nanoporous samples. Here, the emergence of the Ni (111) reflection starts at 700 °C. For s‐LSTN, no signals of the nanoparticles are detected, due to the higher intensity of the perovskite reflections. Upon exsolution, the porosity of n‐LSTN was completely retained (Table 1, **Figure** **3**, and Figure S7, Supporting Information) with only a light shrinkage after treatment at 900 °C.

Our findings so far indicate that matrix nanostructuring strongly influences the extent of reduced material. To determine whether it plays a role also in the nanoparticle growth process we combined electron microscopy and SAXS investigations. In the sintered system, SEM analyses (Figure 3a–c) showed the emergence of sporadic Ni nanoparticles (15 nm) at 700 °C. Their population and size were significantly enhanced after treatment at 800 °C (22 nm) and 900 °C (30 nm), with a gradual Ni depletion from the surface layer (Figure 3d).

Due to the nanostructured character of n‐LSTN, the presence of Ni nanoparticles could be unambiguously detected only by TEM‐EDX (Figure 3e–h). Spheroidal Ni segregations of polydispersed size from 12 to 36 nm are formed by increasing the exsolution temperature. Such growth is accompanied by clear migration of Ni species from the matrix (Figure 3i). More precise information on the exsolution process of n‐LSTN was gathered by in situ TEM analyses (Figure 3j–m). Here it can be clearly observed that the segregation process of Ni starts at a temperature as low as 500 °C, in agreement with XANES results. More interestingly, these measurements show that for T > 700 °C the growth of existing nanoparticles is favored over the formation of new ones. This suggests that growth, fostered by Ni migration and accumulation towards already existing nuclei, as already seen in Figure 3m, is preferred over secondary nucleation at high temperatures.

The main limit of TEM‐EDX mapping in analyzing the nanoparticle morphology is that it provides only an indication of the size of the metal segregation without showing the real size of the metal nanoparticle. Direct and unambiguous information on nanoparticle size is instead provided by SAXS measurements. As SAXS is directly sensitive to the electron density variation of nanostructures,^[^ ^44^, ^45^, ^46^ ^]^ it is particularly suitable to detect metal nanoparticles dispersed in a metal oxide matrix.

**Figure 4:**
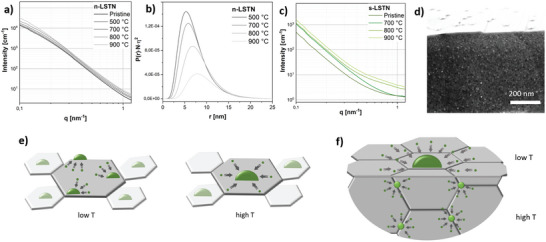
SAXS curves of n‐LSTN exsolved at a) different temperatures and b) respective size distribution of the exsolved Ni nanoparticles; c) SAXS curves of s‐LSTN exsolved at different temperatures; d) Cross‐sectional SEM of s‐LSTNR900 showing the presence of endogenous Ni nanoparticles; e) Schematic representation of the exsolution mechanism from n‐LSTN at low and high temperature; f) Schematic representation of the exsolution mechanism from s‐LSTN at low and high temperature.

SAXS measurements on the nanostructured system exsolved at different temperatures are shown in **Figure** **4**a. The curves resemble a typical distribution of polydispersed nanoobjects on a supporting matrix.^[^ ^47^, ^48^, ^49^ ^]^ The scattering intensity increased at low q‐values by increasing the exsolution temperature. The average radius (*R*) and the number density (N*η* ^2^) of the Ni nanoparticles were extracted from the SAXS curves using a model of spherical nanoparticles with a lognormal distribution (Table 1, Figures S7 and S8, Supporting Information).^[^ ^47^ ^]^

From Figure 4b it is clear that the nanoparticles have a broad size distribution with an average diameter growing from 14 to 20 nm, in quite good agreement with the EDX values. The nanoparticle density per volume unit progressively decreases with the increase in exsolution temperature following the nucleation theory.^[^ ^50^ ^]^ Interestingly, this behavior is the same found in exsolved sintered systems in previous studies using SEM^[^ ^51^, ^52^ ^]^ thus indicating that the growth mechanism of surface exsolved nanoparticles is independent of the nanostructure of the parental matrix. We explain this exsolution mechanism as follows: at low temperatures (500 °C), the poor oxygen release and the sluggish ion kinetics allow the formation of many nuclei, which obviously grow limitedly. As the temperature increases, the larger oxygen release favors the formation of large aggregates of metallic Ni. Due to the faster mobility at these conditions, Ni atoms diffuse preferentially to already existing nuclei, favoring their growth instead of nucleating to form additional nanoparticles (Figure 4e), in good agreement with the in situ TEM results.

With respect to the nanoporous samples, the SAXS curves of the sintered materials (Figure 4c) look different and display an intensity increase proportional to the enhancement of the exsolution temperature at high *q*‐values. This signal represents the progressive formation of scattering bodies of a few nanometers and therefore, cannot be originated from the large surface nanoparticles observed by SEM. Precise analysis of the sample treated at 900 °C (Figure S9, Supporting Information), using the model of randomly packed spheres,^[^ ^53^, ^54^ ^]^ indicates the presence of nanostructures of ≈ 8 nm resembling endogenous Ni nanoparticles formed in the sintered LSTN matrix, as also cross‐sectional SEM confirmed (Figure 4d). Because the scattering contribution of these small nanoparticles is dominating the scattering intensity, the larger ones on the surface cannot be observed by SAXS. So far, the general understanding was that the formation of endogenous nanoparticles is triggered only if particular boundary conditions, such as low cation mobility (i.e., low A‐site deficiency), long reduction time, and high concentration of exsolvable cations (e.g., 40 at.%) are fulfilled.^[^ ^55^ ^]^ Our results show that these conditions are not necessary and that confined Ni nanoparticles form contemporarily to the surface counterparts, without any particular boundary condition. This opens new prospects for the understanding of the exsolution process, as it shows that the Ni ions do not necessarily need to reach the perovskite surface to get reduced and nucleate, as previously reported.^[^ ^56^ ^]^ Instead, the electrons formed by the oxygen release reaction (Equation (2a)) can diffuse throughout the material and fully reduce also lattice Ni ions. Obviously, nucleation is favored at the surface, because it is the portion of the material in direct contact with the reducing agent and with the higher ionic mobility. This is the reason why at 700 °C s‐LSTN shows surface nanoparticles but no endogenous ones. However, at higher temperatures when the larger oxygen release increases the electron concentration, particles form progressively in the bulk as well. As however the mobility is much lower and the constraint much higher than at the surface, nucleation is favored over growth, thus explaining why the population of nanoparticles is much larger in the bulk than at the surface (Figure 4f), in agreement with the previous studies.^[^ ^55^ ^]^

### 2.3 Catalytic Performance

**Figure 5:**
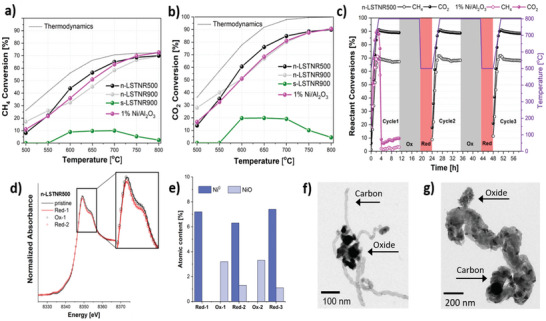
a) CH_4_ conversion and b) CO_2_ conversion on the different Ni‐based catalysts during the biogas dry reforming, as a function of reaction temperature; c) CH_4_ and CO_2_ conversion on n‐LSTNR500 and 1% Ni/Al_2_O_3_ during three catalytic cycles separated by catalyst regeneration steps (oxidation at 800 °C and reduction at 500 °C); d) Ni‐XANES spectra of n‐LSTNR500 after each oxidation and reduction step and corresponding atomic amounts of Ni and NiO e) obtained by linear combination fitting of the XANES spectra; TEM images of the spent n‐LSTNR500 f) and 1% Ni/Al_2_O_3_ g) after the catalytic cycles. Thermodynamic equilibrium values in (a) and (b) calculated using Aspen Plus (CH_4_:CO_2_ = 1.5:1 and *p* = 1 atm) are represented by the dotted lines.

Sintered and nanostructured exsolved materials were tested for the biogas dry reforming reaction and their catalytic performance was compared to a commercial 1 wt.% Ni/Al_2_O_3_ catalyst. In addition to the presence of the exsolved Ni nanoparticles, the A‐site deficient character of the support matrix can exert a catalytic function. The oxygen vacancies formed to compensate for the missing A‐site cations are ideal adsorption sites for CO_2_^[^ ^57^, ^58^ ^]^ and therefore contribute to its conversion. Initial activity experiments (**Figure** **5**a,b) revealed similar CH_4_ and CO_2_ conversion values for the nanoporous catalysts, compared to the commercially available 1 % Ni/Al_2_O_3_ reference, with up to 70 % CH_4_ conversion and 90 % CO_2_ conversion at temperatures beyond 750 °C. The sintered catalyst (s‐LSTNR900) not only displayed much lower conversion rates of merely 10–20 %, but it also deactivated during the experiment, which is expected to be a result of the covering of the Ni nanoparticles with coke during the catalytic testing. Since the sintered catalyst has a minuscule available surface area that does not exceed 1 m^2^ g^−1^, its decreased catalytic activity can be attributed to the limited availability of surface active sites for the reaction to occur. These low‐in‐population surface active sites can also be easily entirely blocked even by a limited amount of deposited coke during the experiment. As such, it is likely that the observed decrease in activity for s‐LSTNR900 in Figure 5a,b is a result of the time spent under the reactant stream.

The CH_4_/CO_2_ ratio used herein (1.5, simulating a typical biogas composition) also has an important effect on the reaction (as CO_2_ is the limiting reactant). It has been previously reported^[^ ^59^, ^60^ ^]^ that a higher CH_4_/CO_2_ ratio (as in our case) can result in lower CH_4_ conversion and CO production, and to higher CO_2_ conversion and H_2_ production, as well as an increase in coke deposition and deactivation. As such, the H_2_/CO ratio (or rather the selectivity of the H_2_ product over CO) is promoted at a higher CH_4_/CO_2_ ratio. Therefore, the higher values for CO_2_ conversion compared to CH_4_ conversion (Figure 5a,b) and the mostly higher than unity H_2_/CO ratio (Figure S10, Supporting Information) can be explained by this higher than equimolar CH_4_/CO_2_ feed ratio.

Among all of the materials, n‐LSTNR500 showed the best performance between 600–700 °C, which could be attributed to the larger concentration and smaller size of Ni nanoparticles, the larger matrix porosity, as well as to the presence of nickel ions still incorporated in the perovskite matrix, which induces the formation of oxygen defects sites.^[^ ^61^ ^]^ n‐LSTNR900 showed higher activity at the low‐temperature range (500–550 °C) since this catalyst initially contained a much higher amount of metallic Ni^0^ (Table 1). The fact though that n‐LSTNR500 surpasses n‐LSTNR900 in catalytic activity above this temperature can be attributed to a further Ni reduction under the CH_4_‐rich reaction atmosphere, something that has also been observed and studied in detail in other works.^[^ ^62^, ^63^, ^64^ ^]^ 1% Ni/Al_2_O_3_ had a similar performance to n‐LSTNR900 above 550 °C, whereas in all catalysts the H_2_/CO ratio was close to 1 (Figure S10a, Supporting Information).

In order to verify whether the well‐known exsolved nanoparticle stability (i.e., socketing) and nanoparticle regenerability also apply to nanoporous systems, thus yielding high‐performance catalysts, the n‐LSTNR500 system was further employed for long‐term time‐on‐stream tests in a total of three cycles (Figure 5c). In order to examine the catalyst regenerability, regeneration steps (i.e., oxidation at 800 °C and reduction at 500 °C) were introduced between each catalytic cycle. After the introduction of biogas at 500 °C, it can be seen that CO_2_ and CH_4_ conversion rates rapidly increase with rising temperature (due to the endothermic nature of the reaction), reaching maximum values of ≈90 % CO_2_ conversion and 70 % CH_4_ conversion at 800 °C (similarly to the previous activity experiments). Once 800 °C is reached, the nanoporous n‐LSTNR500 catalyst exhibits remarkably high stability over 8 h under time‐on‐stream, as only 3 % conversion loss was recorded within the first 5 h. This is even more impressive when considering the harsh reaction conditions used, that is, a high CH_4_/CO_2_ feed ratio of 1.5, which is favorable to catalyst deactivation via coking.^[^ ^59^, ^60^ ^]^ Most notably following catalyst regeneration, the catalytic performance was entirely recovered in both 2^nd^ and 3^rd^ cycles, proving the regenerability of such a nanostructured exsolved catalytic system. Furthermore, the nanostructured catalyst yielded syngas with a constant H_2_/CO molar ratio close to 1 during all three cycles (Figure S10b, Supporting Information).

When the commercial 1 % Ni/Al_2_O_3_ catalyst underwent the same long‐term stability test, it behaved differently. Although during temperature ramping (from 500 to 800 °C) the catalytic performance of 1 % Ni/Al_2_O_3_ was comparable to the exsolved n‐LSTNR500 system (even the H_2_/CO ratio was close to 1), it displayed a sharp loss of CH_4_ and CO_2_ conversion (down to less than 10 %) shortly after reaching the final temperature of 800 °C (≈2 h), which can be attributed to a severe catalyst deactivation via coke deposition and the encapsulation of Ni nanoparticles in it. These results clearly show the outstanding performance of nanoporous exsolved materials as high‐temperature reforming catalysts, providing high long‐term stability and full performance recovery following regeneration.

In order to verify whether and to what extent n‐LSTNR500 was regenerated, we demonstrated the redox cyclability of Ni via XANES spectroscopy (Figure 5d and Figure S11, Supporting Information). Because it was not possible to sample the catalyst during the long‐term stability test, we reproduced the regeneration conditions on a second reactor in the lab. All the Ni spectra in the oxidized state differ from the reduced ones by a small but distinct intensity decrease of the white line and the pre‐edge feature. Interestingly, all the curves collected in the reduced and oxidized state superposed, suggesting high reproducibility of the regeneration process. In particular, LCF analysis (Figure 5e and Figure S12, Supporting Information) showed that in each reduction event a constant amount of ≈7 % of metallic Ni was produced. The majority of Ni was successfully reincorporated into the perovskite matrix following oxidation, although a certain amount of NiO was detected after the first and second oxidation events. The presence of NiO can be ascribed to incomplete dopant reincorporation probably because of a too‐low oxidation temperature.^[^ ^65^, ^66^ ^]^ In our case, however, the main objective of regaining the catalyst's activity following regeneration was nevertheless achieved.

In order to assess the structure and morphology variation of the spent catalysts as well as the presence of carbon deposits, TEM analyses were performed (Figure 5f,g). Microscopy analysis shows surface reorganization of the exsolved catalyst n‐LSTNR500, but only the relatively limited formation of thin carbon whiskers. The majority of the catalyst surface appears free from carbon deposits, thus enabling continuous reactant conversions. The carbon whiskers that do form, appear to grow on top of the Ni nanoparticles, rather than detaching them from their support, as a result of the strong metal‐support interaction. Moreover, many defects can be observed on the thin chain‐like carbon whiskers, which can be attributed to the high oxygen lability of the nanostructured perovskite support, as labile oxygen species can cause a discontinued growth of the carbon filaments.^[^ ^67^, ^68^ ^]^ No Ni nanoparticle encapsulation was observed for the exsolved nanostructured system, likely due to the socketing of the exsolved Ni nanoparticles in the perovskite matrix.^[^ ^69^ ^]^ On the contrary, coke deposits form extensively on the commercial Ni/Al_2_O_3_ catalyst, leading to much more dense carbon nanostructures (thick‐walled carbon nanotubes) leading to catalyst deactivation (Figure 5c).

**Scheme 1:**
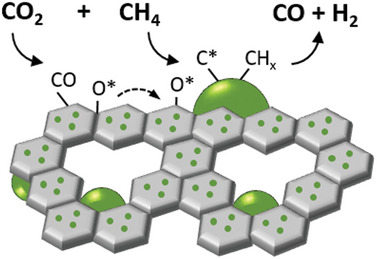
Representation of the catalytic mechanism over the n‐LSTNR500 catalyst. The green semicircles and the green dots indicate Ni nanoparticles and Ni dopants in the nanoporous matrix, respectively. CH_4_ chemisorption and decomposition into adsorbed carbon (C* or CH_x_) species as well as H_2_ adsorption take place over the exposed metallic Ni surface. Instead, CO_2_ dissociation occurs primarily on the perovskite oxygen defect sites. The labile lattice oxygen species (O*) resulting from CO_2_ dissociation can then migrate to oxidize adsorbed carbon species on the Ni surface into CO.

A proposed catalytic reaction mechanism over the nanoporous exsolved n‐LSTNR500 system can be found in **Scheme** **1**. It is proposed, that during biogas dry reforming, CH_4_ chemisorption and decomposition into adsorbed carbon (C* or CH_x_) species and H_2_ takes place over the exposed metallic Ni surface, whereas CO_2_ activation and dissociation take place on the metal‐support interface or the adjacent support oxygen defect sites.^[^ ^70^, ^71^, ^72^ ^]^ The population of the defect sites can be enhanced via the presence of nickel ions solubilized in the perovskite matrix.^[^ ^61^ ^]^ The labile surface oxygen species resulting from CO_2_ dissociation can then migrate to oxidize adsorbed carbon species on the Ni surface into CO.^[^ ^70^, ^71^ ^]^ Oxygen defect sites, especially those adjacent to metallic Ni, are very important during the reaction, as they enhance CO_2_ adsorption (which in our case also acts as the limiting reactant) and promote the migration of labile oxygen species, thereby favoring the gasification of adsorbed carbon moieties on the Ni surface and thus mitigating carbon deposition and polymerization.^[^ ^61^, ^70^, ^71^ ^]^

## 3 Conclusion

In this paper, we provided a detailed description of the exsolution process of Ni from nanoporous La_0.52_Sr_0.28_Ti_0.94_Ni_0.06_O_3±*δ*_ and its application as an effective, stable catalyst for the biogas dry reforming reaction. We showed that the reduction mechanism from nanoporous matrices is faster and more extensive than from sintered counterparts, as a consequence of the much higher oxygen mobility and its larger release. By using SAXS as an alternative, effective method to study the nanoparticle formation process, we demonstrated that Ni nanoparticles on nanoporous matrices grow on surfaces with the same mechanism as that of sintered ones. Differently from what is known so far, this method also showed that in sintered materials endogenous nanoparticles always form at high temperatures (T > 800 °C), without any particular boundary conditions, thus clearly indicating that Ni ions do not necessarily need to reach the perovskite surface to get reduced and nucleate. Biogas dry reforming tests evidenced the superior catalytic activity of nanoporous exsolved systems compared to sintered counterparts. Irrespective of the highly defective matrix grain texture, nanoparticles on nanoporous LSTN showed high long‐term catalytic stability and multiple, complete regeneration, largely surpassing the performance of commercial Ni/Al_2_O_3_ during time‐on‐stream tests.

The design of parental oxides with specific nanostructure and nanoporosity represents therefore an appealing strategy for the further development of exsolved catalysts by exploiting the unconventional ion mobility at the nanoscale.

## 4 Experimental Section

### Chemicals

Strontium nitrate (99 %, Acros Organics), titanium‐(IV) isopropoxide (97 %, Alfa Aesar), lanthanum(III) nitrate hexahydrate (99.9 %, Alfa Aesar), nickel nitrate hexahydrate (97 %, Sigma‐Aldrich) and anhydrous citric acid (99.6 %, Acros Organics), and glycerol (99 %, Alfa Aesar) were used as received without further purification. The commercial 1 % Ni/Al_2_O_3_ was purchased by Riogen Inc., NJ, USA.

### Materials Synthesis and Characterization

The nanoporous perovskite oxide was prepared according to the chelate complex route as previously reported by Kayaalp et al.^[^ ^31^, ^32^, ^73^ ^]^ In the first step, 5.11 mmol of titanium(IV)isopropoxide was added to 11.9 mL of glycerol and stirred at room temperature for 30 min. After the addition of citric acid (40.7 mmol), the reaction mixture was heated up to 60 °C in an oil bath and maintained for 1 h until complete dissolution. Subsequently, 1.52 mmol Sr(NO_3_)_2_ dissolved in 1 mL deionized water, 2.82 mmol La(NO_3_)_3_∙6H_2_O, and 0.33 mmol Ni(NO_3_)_2_∙6H_2_O were successively added in 15 min intervals to the solution under continuous stirring and equilibrated for 2 h. Further temperature increase to 130 °C resulted in a covalent network via polyesterification of the chelating agents. The obtained polymer gel was then calcined under static air with a heating ramp of 2 °C∙min^−1^ at 800 °C for 2 h. In order to remove the organic content an intermediate step at 400 °C for 2 h was realized before reaching the final temperature. To produce highly dense bulk material the LSTN powders were pressed into pellets of 1 g and sintered in the air with a heating ramp of 10 °C∙min^−1^ at 1200 °C for 10 h.

Exsolution treatments of the perovskite oxide powders were carried out in a U‐shaped quartz tube with a 4 mm inner diameter filled with 0.25 g of the sample and coupled with a Cirrus2 MKS mass spectrometer. 5 % H_2_/N_2_ (volume ratio 5/95) was chosen as reducing atmosphere with a flow rate of 1.2 l∙h^−1^. The samples were heated with a ramp of 10 °C min^−1^ up to the target temperatures indicated in the main text and kept there for 5 h before cooling down to room temperature. The materials were characterized shortly after the reduction process and kept in closed glass vials to avoid aging.

Synchrotron XRD measurements of the pristine materials were carried out at the P02.1 beamline of PETRA‐III at DESY, Hamburg. The powder samples were packed into quartz‐glass capillaries with an interior diameter of 0.99 nm. The capillary was rotated in the Debye‐Scherrer geometry and monochromatically irradiated with an energy of about 60 keV (*λ* =  0.20735 Å). Calibration of the wavelength was done with silicon as an external standard. By using Fit2D software the obtained raw diffraction data were integrated into 1D powder patterns.^[^ ^74^ ^]^ GISAS was used to perform Rietveld refinements.^[^ ^75^ ^]^

The composition and lattice parameters of the samples could be refined in the cubic space group *Pm‐3m*. The structural solutions of the diffractograms were based on the database entry of La_0.33_Sr_0.67_TiO_3_ (ICSD #247635) modified with 6% Ni occupation on the B‐site, with the space group *Pm*‐3*m*. Thus, a general composition of La_0.52_Sr_0.28_Ti_0.94_Ni_0.06_O_3+*δ*_ has been obtained for all pristine samples. The XRD measurements of the exsolved materials were carried out at beamline P08 of PETRA‐III at DESY Hamburg (Germany). The powder samples were packed into glass capillaries with an internal diameter of 0.99 nm and then monochromatically irradiated with an energy of about 18 keV (*λ* = 0.68987 Å) in the Debye–Scherrer geometry. A 6‐Circle diffractometer Kohzu NZD‐3 with a closed Eulerian cradle was used to obtain the diffraction data. Calibration of the wavelength was carried out with lanthanum hexaboride as an external standard. The raw diffraction data were integrated into one‐dimensional powder patterns. The full width at half maximum (FWHM) of the reflection of the highest intensity was used in order to calculate the average crystallite sizes by applying the Scherrer equation.^[^ ^76^ ^]^

The SAXS experiments have been performed at the beamline P62 of PETRA III at DESY, Hamburg. The powder samples were packed into glass capillaries with an internal diameter of 0.99 nm and then monochromatically irradiated with an energy of about 7 keV (*λ* = 1.7712 Å) and a pinhole collimation was used. The raw data have been corrected for the incoming beam flux, transmission, and scattering background. The scaling to absolute differential scattering cross section has been done using a pre‐calibrated glassy carbon sample. The reduced scattering patterns of the exsolved materials were analyzed using the software package SASfit^[^ ^77^ ^]^ and Scatter.^[^ ^78^ ^]^

The nitrogen physisorption isotherms were recorded at 77 K using a Quadrasorb SI‐MP by Quantachrome. Samples were outgassed with a Masterprep Degasser (Quantachrome Corp.) at 120 °C for 12 h. In the range of p/p^0^ = 0.07–0.3 specific surface areas were calculated with the Brunauer–Emmett–Teller (BET) method.^[^ ^79^ ^]^ Pore size distribution was determined with the NLDFT method^[^ ^79^ ^]^ applying the model for silica cylindrical pores on the adsorption branch by utilizing the Quantachrome ASiQWin software.

TEM measurements were performed on a JEOL JEM 2200 FS operating at 200 kV equipped with two CEOS Cs correctors (CETCOR, CESCOR), a JEOL JED‐2300 Si(Li) EDX (energy dispersive X‐ray spectroscopy) detector, a Gatan 4K UltraScan 1000 camera, and a HAADF (high angle annular dark field) detector. The sample was ground into fine powder, which was dispersed in ethanol using an ultrasonic bath and subsequently dropped on a carbon‐coated 400 mesh TEM grid. A filter paper and drying in air at room temperature were used to remove the excess solvent. EDX spectra and elemental mapping were carried out using 256 × 256 pixels (pixel size of 0.7 nm) and a dwell time of 0.5 ms pixel^−1^ (corrected for dead time) with 30 cycles. These measurements were repeated at least on three different positions for each sample to ensure statistical relevance. In situ TEM measurements were performed under vacuum (3e‐5 Pa). The targeted temperature was reached with a heating ramp of 10 °C∙min^−1^, then held for 30 min before the measurements were performed. Subsequently, the sample environment was cooled down to ambient temperature before it was heated up again to the following, higher temperature. In order to avoid beam damage the acquisition spot was changed every two temperature steps.

XPS data were acquired with a Perkin–Elmer *φ* 5600ci instrument using Al‐K_*α*_ radiation (1486.6 eV), operating at 350 W at a working pressure less than 5·10^−8^ Pa. The instrument calibration was based on the binding energy (BE) of the Au 4f7/2 line at 83.9 eV with respect to the Fermi level. The standard deviation for the BE values was 0.15 eV. The obtained BEs were corrected for charging effects, assigning to the C1s line of adventitious carbon the BE value of 284.6 eV. Survey scans were measured in the 0–1350 eV range (187.5 eV pass energy, 1.0 eV step^−1^, 25 ms step^−1^).

XAS of the Ni K‐edge (8.333 keV) were performed at the P64 beamline at the Deutsches Elektronen Synchrotron (DESY, Hamburg, Germany)^[^ ^80^ ^]^ using a Si (111) double crystal monochromator for the energy scans around the metal absorption edge. The XAS spectra were collected in fluorescence configuration using a PIPS detector. The sample pellets (28 µm thick) were prepared by diluting the powder in cellulose and then covered in Kapton tape before placing them in the beam path. Athena software was used to normalize the obtained XAFS data by subtracting the pre‐ and post‐edge background based on the AUBACK algorithm.^[^ ^81^ ^]^ The collected data were reduced and evaluated using the Demeter package.^[^ ^81^ ^]^ For LCF analysis, spectra of metallic Ni, NiO, and LSTN, that is, the pristine matrix were used as references for all the measured spectra, in the range 8313–8413 eV. The Artemis software was employed to fit the extracted EXAFS functions. Passive electron reduction factor (S02) was obtained from the fit of bulk metallic Ni (S02 = 0.79) and kept constant in the fits for all the samples. The experimental data (k‐range: 3–12 Å^−1^, R‐range: 1.0–2.8 Å) were fitted in R‐space (*k*‐weighting = 2). Since the LCF analysis did not show any contribution of NiO to the experimental spectra, for the modeling of the EXAFS curves only paths of metallic Ni and LSTN were taken into consideration. In the first step, the coordination numbers of the considered path were left free to vary during the fitting, but the resulting values were in all cases corresponding to the bulk ones (6 for Ni–O and 12 for Ni–Ni). Further iterations to improve the overall fit quality were performed with these values kept fixed, and the results are here reported and discussed. The weights of Ni–O and Ni–Ni contributions were forced to sum to 1.

### Catalytic Testing

The dry reforming of biogas was carried out in a quartz tube reactor (I.D. = 0.9 cm) at atmospheric pressure. The catalyst mass used was 100 mg (exsolved perovskites), diluted with 400 mg of quartz sand. The total flow rate was 40 ml min^−1^, with 10 ml min^−1^ of simulated biogas flow (60% CH_4_ and 40% CO_2_ gas mixture, that is, CH_4_/CO_2_ feed ratio of 1.5) and 30 ml min^−1^ Ar flow (used as carrier gas).

The catalytic activity as a function of reaction temperature (temperature step experiments) was assessed via experimental protocol #1. At first, the reactor temperature was elevated to 500 °C under Ar flow, followed by the introduction of the feed gas mixture. Then, it was stepwise increased using 50 °C steps up to 800 °C, remaining for 30 min at each temperature step. The catalytic stability and regenerability were evaluated via experimental protocol #2. The reactor temperature was initially raised to 500 °C under Ar flow (Cycle 1). After that, the feed gas mixture was introduced, and the temperature was steadily increased by 50 °C every 30 min up to 800 °C. Once the final temperature of 800 °C was reached, the catalyst was kept under the reactant stream at this temperature for another 8 h. For catalyst regeneration, oxidation was first carried out under 20% O_2_/Ar at 800 °C for 8 h (to induce nanoparticle reincorporation into the perovskite host), followed by cooling under Ar to 500 °C and then reduction at this temperature under 5% H_2_/Ar for 5 h (to induce re‐exsolution of metallic Ni nanoparticles). After the Ar purge, the reactant feed gas mixture was reintroduced (Cycles 2 and 3) following the previous steps as in Cycle 1.

Regarding the 1% Ni/Al_2_O_3_ reference, it was first calcined at 800 °C for 8 h under static air and then in situ reduced in the catalytic reactor under H_2_ flow at 800 °C for 1 h prior to catalytic testing. The reactor was then left to cool under Ar at the starting temperature of 500 °C.

The reaction products in the gas phase were analyzed via online gas chromatography in an Agilent GC‐systems‐7890A with He as carrier gas and two columns in parallel, Agilent J&W HP Plot‐Q (19095‐Q04, 30 m length, 0.530 mm I.D.) and Agilent J&WHP‐Molesieve (19095P‐MSO, 30m length, 0.530 mm I.D.), equipped with TCD and FID detectors. The conversions for methane and carbon dioxide, the yields for hydrogen and carbon monoxide, as well as the H_2_/CO molar ratio, were determined according to the following equations:

(3) $$X_{CH_{4}}=\frac{F_{CH_{4,in}}-F_{CH_{4,out}}}{F_{CH_{4,in}}}\times 100$$

(4) $$X_{CO_{2}}=\frac{F_{CO_{2,\;in}}-F_{CO_{2,\;out}}}{F_{CO_{2,\;in}}}\times 100$$

(5) $$Y_{H_{2}}=\frac{F_{H_{2,\;out}}}{2\times F_{CH_{4,in}}}\times 100$$

(6) $$Y_{CO}=\frac{F_{CO,out}}{F_{CH_{4,in}}+F_{CO_{2,\;in}}}\times 100$$

(7) $$H_{2}/CO=\frac{F_{H_{2,\;out}}}{F_{CO,out}}$$

### Statistical Analysis

In the present work, all surface particle sizes were determined based on electron microscopy. In the case of s‐LSTN, SEM images of plane surfaces were chosen whereas for n‐LSTN areas of clearly definable segregations of EDX mappings were investigated. Subsequent individual particle sizes examination was carried out by using the commercial ImageJ software. Due to varying particle populations, an average particle size value was obtained from the highest number possibly examined. Endogenous particle size determination by SAXS as well as the corresponding variance derived from the fitting function as given in the main part. Both bond lengths and the respective standard deviations via EXAFS analysis were deduced from the fitting procedures as particularized in the Experimental Section.

## Conflict of Interest

The authors declare no conflict of interest.

## Supporting information

Supporting Information

## Data Availability

The data that support the findings of this study are available on request from the corresponding author. The data are not publicly available due to privacy or ethical restrictions.

## References

[1] M. Meinshausen , N. Meinshausen , W. Hare , S. C. B. Raper , K. Frieler , R. Knutti , D. J. Frame , M. R. Allen , Nature 2009, 458, 1158. 19407799 10.1038/nature08017

[2] T. Nevzorova , V. Kutcherov , Energy Strategy Rev. 2019, 26, 100414.

[3] Q. Sun , H. Li , J. Yan , L. Liu , Z. Yu , X. Yu , Renewable Sustainable Energy Rev. 2015, 51, 521.

[4] S. Jung , J. Lee , D. H. Moon , K.‐H. Kim , E. E. Kwon , Renewable Sustainable Energy Rev. 2021, 143, 110949.

[5] N. D. Charisiou , G. Siakavelas , K. N. Papageridis , A. Baklavaridis , L. Tzounis , D. G. Avraam , M. A. Goula , J. Nat. Gas Sci. Eng. 2016, 31, 164.

[6] M. A. Goula , N. D. Charisiou , G. Siakavelas , L. Tzounis , I. Tsiaoussis , P. Panagiotopoulou , G. Goula , I. V. Yentekakis , Int. J. Hydrogen Energy 2017, 42, 13724.

[7] B. Abdullah , N. A. Abd Ghani , D.‐V. N. Vo , J. Cleaner Prod. 2017, 162, 170.

[8] M. A. Goula , N. D. Charisiou , K. N. Papageridis , A. Delimitis , E. Pachatouridou , E. F. Iliopoulou , Int. J. Hydrogen Energy 2015, 40, 9183.

[9] C. M. Damaskinos , J. Zavašnik , P. Djinović , A. M. Efstathiou , Appl. Catal., B 2021, 296, 120321.

[10] N. Charisiou , S. Douvartzides , G. Siakavelas , L. Tzounis , V. Sebastian , V. Stolojan , S. Hinder , M. Baker , K. Polychronopoulou , M. Goula , Catalysts 2019, 9, 676.

[11] U. Guharoy , T. R. Reina , J. Liu , Q. Sun , S. Gu , Q. Cai , J. CO2 Util. 2021, 53, 101728.

[12] A. I. Tsiotsias , N. D. Charisiou , V. Sebastian , S. Gaber , S. J. Hinder , M. A. Baker , K. Polychronopoulou , M. A. Goula , Int. J. Hydrogen Energy 2022, 47, 5337.

[13] L. Wu , X. Xie , H. Ren , X. Gao , Mater Today Proc 2021, 42, 153.

[14] N. D. Charisiou , G. Siakavelas , L. Tzounis , V. Sebastian , A. Monzon , M. A. Baker , S. J. Hinder , K. Polychronopoulou , I. V. Yentekakis , M. A. Goula , Int. J. Hydrogen Energy 2018, 43, 18955.

[15] Z. Bian , Z. Wang , B. Jiang , P. Hongmanorom , W. Zhong , S. Kawi , Renewable Sustainable Energy Rev. 2020, 134, 110291.

[16] K. Kousi , D. Neagu , L. Bekris , E. Calì , G. Kerherve , E. I. Papaioannou , D. J. Payne , I. S. Metcalfe , J. Mater. Chem. A 2020, 8, 12406.

[17] K. Xie , Y. Xiao , Angew. Chem., Int. Ed. 2022, 61, e202113079. 10.1002/anie.202113079 34676642

[18] S. P. Padi , L. Shelly , E. P. Komarala , D. Schweke , S. Hayun , B. A. Rosen , Catal. Commun. 2020, 138, 105951.

[19] J. H. Kim , J. K. Kim , J. Liu , A. Curcio , J.‐S. Jang , I.‐D. Kim , F. Ciucci , W. Jung , ACS Nano 2021, 15, 81. 33370099 10.1021/acsnano.0c07105

[20] D. Burnat , R. Kontic , L. Holzer , P. Steiger , D. Ferri , A. Heel , J. Mater. Chem. A 2016, 4, 11939.

[21] P. Steiger , D. Burnat , H. Madi , A. Mai , L. Holzer , J. Van Herle , O. Kröcher , A. Heel , D. Ferri , Chem. Mater. 2019, 31, 748.

[22] D. Neagu , V. Kyriakou , I.‐L. Roiban , M. Aouine , C. Tang , A. Caravaca , K. Kousi , I. Schreur‐Piet , I. S. Metcalfe , P. Vernoux , M. C. M. van de Sanden , M. N. Tsampas , ACS Nano 2019, 13, 12996. 31633907 10.1021/acsnano.9b05652

[23] H. Arandiyan , Y. Wang , J. Scott , S. Mesgari , H. Dai , R. Amal , ACS Appl. Mater. Interfaces 2018, 10, 16352. 29522310 10.1021/acsami.8b00889

[24] B. Hua , M. Li , Y. F. Sun , Y. Q. Zhang , N. Yan , J. Chen , T. Thundat , J. Li , J. L. Luo , Nano Energy 2017, 32, 247.

[25] B. E. Kayaalp , Y. J. Lee , A. Kornowski , S. Gross , M. D'Arienzo , S. Mascotto , RSC Adv. 2016, 6, 90401.

[26] P. Hongmanorom , J. Ashok , P. Chirawatkul , S. Kawi , Appl. Catal., B 2021, 297, 120454.

[27] J. Liang , Z. Liang , R. Zou , Y. Zhao , Adv. Mater. 2017, 29, 1701139. 10.1002/adma.201701139 28628246

[28] J. Scholz , A. Garbujo , B. Kayaalp , K. Klauke , A. Glisenti , S. Mascotto , Inorg. Chem. 2019, 58, 15942. 31710214 10.1021/acs.inorgchem.9b02460

[29] M. T. Elm , J. D. Hofmann , C. Suchomski , J. Janek , T. Brezesinski , ACS Appl. Mater. Interfaces 2015, 7, 11792. 25984884 10.1021/acsami.5b01001

[30] P. Hartmann , T. Brezesinski , J. Sann , A. Lotnyk , J.‐P. Eufinger , L. Kienle , J. Janek , ACS Nano 2013, 7, 2999. 23514447 10.1021/nn400255w

[31] E. Poffe , H. Kaper , B. Ehrhardt , L. Gigli , D. Aubert , L. Nodari , S. Gross , S. Mascotto , ACS Appl. Mater. Interfaces 2021, 13, 25483. 34006105 10.1021/acsami.1c02281

[32] B. Kayaalp , S. Lee , K. Klauke , J. Seo , L. Nodari , A. Kornowski , W. C. Jung , S. Mascotto , Appl. Catal., B 2019, 245, 536.

[33] B. Kayaalp , K. Klauke , M. Biesuz , A. Iannaci , V. M. Sglavo , M. D'Arienzo , H. Noei , S. Lee , W. Jung , S. Mascotto , J. Phys. Chem. C 2019, 123, 16883.

[34] K. Klauke , B. Kayaalp , M. Biesuz , A. Iannaci , V. M. Sglavo , M. D'Arienzo , S. Lee , S. Jongsu , W. Jung , S. Mascotto , ChemNanoMat 2019, 5, 948.

[35] D. Neagu , G. Tsekouras , D. N. Miller , H. Ménard , J. T. S. Irvine , Nat. Chem. 2013, 5, 916. 24153368 10.1038/nchem.1773

[36] R. Meyer , R. Waser , J. Helmbold , G. Borchardt , J. Electroceram. 2002, 9, 101.

[37] A. Demont , S. Abanades , E. Beche , J. Phys. Chem. C 2014, 118, 12682.

[38] A. I. Tsiotsias , B. Ehrhardt , B. Rudolph , L. Nodari , S. Kim , W. Jung , N. D. Charisiou , M. A. Goula , S. Mascotto , ACS Nano 2022, 16, 8904. 35709497 10.1021/acsnano.1c11111

[39] A. M. Beale , M. Paul , G. Sankar , R. J. Oldman , C. R. A. Catlow , S. French , M. Fowles , J. Mater. Chem. 2009, 19, 4391.

[40] P. Steiger , I. Alxneit , D. Ferri , Acta Mater. 2019, 164, 568.

[41] R. J. Woolley , B. N. Illy , M. P. Ryan , S. J. Skinner , J. Mater. Chem. 2011, 21, 18592.

[42] N. S. Marinković , K. Sasaki , R. R. Adžić , Zast. Mater. 2016, 57, 101.

[43] A. M. Beale , B. M. Weckhuysen , Phys. Chem. Chem. Phys. 2010, 12, 5562. 20379576 10.1039/b925206a

[44] L. Tripaldi , E. Callone , M. D'Arienzo , S. Dirè , L. Giannini , S. Mascotto , A. Meyer , R. Scotti , L. Tadiello , B. Di Credico , Soft Matter 2021, 17, 9434. 34611686 10.1039/d1sm01085a

[45] E. Gericke , D. Wallacher , R. Wendt , G. Greco , M. Krumrey , S. Raoux , A. Hoell , S. Mascotto , J. Phys. Chem. Lett. 2021, 12, 4018. 33878272 10.1021/acs.jpclett.1c00557

[46] J. Scholz , B. Kayaalp , A. Juhl , D. Clemens , M. Fröba , S. Mascotto , ACS Energy Lett. 2018, 3, 387.

[47] L. B. Kiss , J. Söderlund , G. A. Niklasson , C. G. Granqvist , Nanotechnology 1999, 10, 25.

[48] B. Di Credico , I. Tagliaro , E. Cobani , L. Conzatti , M. D'Arienzo , L. Giannini , S. Mascotto , R. Scotti , P. Stagnaro , L. Tadiello , Nanomaterials 2019, 9, 46. 10.3390/nano9010046 PMC6359008 30602665

[49] M. D'Arienzo , S. Diré , V. Masneri , D. Rovera , B. Di Credico , E. Callone , S. Mascotto , A. Pegoretti , F. Ziarelli , R. Scotti , ACS Appl. Nano Mater. 2018, 1, 3817.

[50] W. T. Wallace , B. K. Min , D. W. Goodman , Top. Catal. 2005, 34, 17.

[51] S.‐K. Otto , K. Kousi , D. Neagu , L. Bekris , J. Janek , I. S. Metcalfe , ACS Appl. Energy Mater. 2019, 2, 7288.

[52] Y. Gao , D. Chen , M. Saccoccio , Z. Lu , F. Ciucci , Nano Energy 2016, 27, 499.

[53] S. Mascotto , D. Wallacher , A. Kuschel , S. Polarz , G. A. Zickler , A. Timmann , B. M. Smarsly , Langmuir 2010, 26, 6583. 20201568 10.1021/la903934r

[54] J. S. Pedersen , J. Appl. Crystallogr. 1994, 27, 595.

[55] K. Kousi , D. Neagu , L. Bekris , E. Papaioannou , I. S. Metcalfe , Angew. Chem., Int. Ed. 2020, 59, 2510. 10.1002/anie.201915140 31804017

[56] K. Kousi , C. Tang , I. S. Metcalfe , D. Neagu , Small 2021, 17, 2006479. 10.1002/smll.202006479 33787009

[57] Y. Pan , C. Liu , D. Mei , Q. Ge , Langmuir 2010, 26, 5551. 20047326 10.1021/la903836v

[58] A. S. Al‐Fatesh , Y. Arafat , S. O. Kasim , A. A. Ibrahim , A. E. Abasaeed , A. H. Fakeeha , Appl. Catal., B 2021, 280, 119445.

[59] M. K. Nikoo , N. A. S. Amin , Fuel Process. Technol. 2011, 92, 678.

[60] E. le Saché , A. Alvarez Moreno , T. R. Reina , Front. Chem. 2021, 9, 223. 10.3389/fchem.2021.672419 PMC8080852 33937208

[61] B. Safavinia , Y. Wang , C. Jiang , C. Roman , P. Darapaneni , J. Larriviere , D. A. Cullen , K. M. Dooley , J. A. Dorman , ACS Catal. 2020, 10, 4070.

[62] N. Bonmassar , M. F. Bekheet , L. Schlicker , A. Gili , A. Gurlo , A. Doran , Y. Gao , M. Heggen , J. Bernardi , B. Klötzer , S. Penner , ACS Catal. 2020, 10, 1102.

[63] C. Batiot‐Dupeyrat , G. A. S. Gallego , F. Mondragon , J. Barrault , J.‐M. Tatibouët , Catal. Today 2005, 107–108, 474.

[64] F. Schrenk , L. Lindenthal , H. Drexler , G. Urban , R. Rameshan , H. Summerer , T. Berger , T. Ruh , A. K. Opitz , C. Rameshan , Appl. Catal., B 2022, 318, 121886.

[65] H. Lv , L. Lin , X. Zhang , Y. Song , H. Matsumoto , C. Zeng , N. Ta , W. Liu , D. Gao , G. Wang , X. Bao , Adv. Mater. 2020, 32, 1906193. 10.1002/adma.201906193 31894628

[66] K. Y. Lai , A. Manthiram , Chem. Mater. 2018, 30, 2838.

[67] A. I. Tsiotsias , N. D. Charisiou , A. AlKhoori , S. Gaber , V. Sebastian , S. J. Hinder , M. A. Baker , K. Polychronopoulou , M. A. Goula , J. CO2 Util. 2022, 61, 102046.

[68] N. D. Charisiou , G. Siakavelas , L. Tzounis , V. Sebastian , A. Monzon , M. A. Baker , S. J. Hinder , K. Polychronopoulou , I. V. Yentekakis , M. A. Goula , Int. J. Hydrogen Energy 2018, 43, 18955.

[69] D. Neagu , T. S. Oh , D. N. Miller , H. Ménard , S. M. Bukhari , S. R. Gamble , R. J. Gorte , J. M. Vohs , J. T. S. Irvine , Nat. Commun. 2015, 6, 8120. 26360910 10.1038/ncomms9120 PMC4579408

[70] C. M. Damaskinos , J. Zavašnik , P. Djinović , A. M. Efstathiou , Appl. Catal., B 2021, 296, 120321.

[71] A. G. S. Hussien , C. M. Damaskinos , A. A. Dabbawala , D. H. Anjum , M. A. Vasiliades , M. T. A. Khaleel , N. Wehbe , A. M. Efstathiou , K. Polychronopoulou , Appl. Catal., B 2022, 304, 121015.

[72] M. Li , Z. Sun , Y. H. Hu , J. Mater. Chem. A 2021, 9, 12495.

[73] B. Kayaalp , S. Lee , L. Nodari , J. Seo , S. Kim , W. C. Jung , S. Mascotto , ACS Appl. Nano Mater. 2020, 3, 11352.

[74] A. P. Hammersley , S. O. Svensson , M. Hanfland , A. N. Fitch , D. Hausermann , High Pressure Res. 1996, 14, 235.

[75] B. H. Toby , J. Appl. Crystallogr. 2001, 34, 210.

[76] A. Monshi , M. R. Foroughi , M. R. Monshi , World J. Nano Sci. Eng. 2012, 2, 154.

[77] I. Breßler , J. Kohlbrecher , A. F. Thünemann , J. Appl. Crystallogr. 2015, 48, 1587. 26500467 10.1107/S1600576715016544 PMC4603274

[78] S. Förster , L. Apostol , W. Bras , J. Appl. Crystallogr. 2010, 43, 639.

[79] M. Thommes , K. Kaneko , A. V. Neimark , J. P. Olivier , F. Rodriguez‐Reinoso , J. Rouquerol , K. S. W. Sing , Pure Appl. Chem. 2015, 87, 1051.

[80] W. A. Caliebe , V. Murzin , A. Kalinko , M. Görlitz , in AIP Conf. Proc., AIP Publishing LLC, 2019, p. 060031.

[81] B. Ravel , M. Newville , J. Synchrotron Radiat. 2005, 12, 537. 15968136 10.1107/S0909049505012719

