# Supplementary material for: Nanoparticle Exsolution from Nanoporous Perovskites for Highly Active and Stable Catalysts
Source: Adv Sci (Weinh). 2023 Jan 22;10(6):2205890. doi: 10.1002/advs.202205890 (PMC9951582; doi:10.1002/advs.202205890)
Supplement: Supplementary file 1 — Supporting Information [file ADVS-10-2205890-s001.pdf]

Supplementary Information

for

Nanoparticle exsolution from nanoporous perovskites for highly active and stable catalysts

Benjamin Rudolph[1], Anastasios I. Tsiotsias[2], Benedikt Ehrhardt[1], Paolo Dolcet[3], Silvia Gross[3,4], Sylvio Haas[5], Nikolaos D. Charisou[2], Maria A. Goula[2], and Simone Mascotto[1*]

[1] Institut für Anorganische und Angewandte Chemie, Universität Hamburg, Martin-Luther-King-Platz, 6, 20146 Hamburg, Germany

[2] Department of Chemical Engineering, University of Western Macedonia, Koila, Kozani, 50100, Greece

[3] Institute for Chemical Technology and Polymer Chemistry, Karlsruhe Institute of Technology, Engesserstrasse 20, 76133 Karlsruhe, Germany

[4] Dipartimento di Scienze Chimiche, Università degli Studi di Padova, via Marzolo 1, 35131 Padova, Italy

[5] Deutsches Elektronen Synchrotron (DESY), Notkestr. 85, 22607 Hamburg, Germany

*Corresponding Author: simone.mascotto@chemie.uni-hamburg.de

Table S1: Summary of the structural and compositional parameters from XRD. Calculated composition, lattice parameter (a) and reliability factors X^2 and $R(F^2)$.

Sample	Calculated composition	Φ [nm]	a [Å]	X^2	$R(F^2)$
s-LSTN	$La_{0.52}Sr_{0.25}Ti_{0.94}Ni_{0.064}O_{2.807}$	-	3.8920 (1)	14.33	0.0134
n-LSTN	$La_{0.53}Sr_{0.27}Ti_{0.95}Ni_{0.05}O_{3.100}$	22 (4)	3.8981 (1)	2.177	0.0171

Table S2: Atomic positions of both n-LSTN and s-LSTN – main phases obtained by Rietveld refinement

**Atom**	**Site**	***x***	***y***	***z***
La1	1a	0	0	0
Sr1	1a	0	0	0
Ti1	1b	0.5	0.5	0.5
Ni1	1b	0.5	0.5	0.5

O1	3c	0	0.5	0.5

Table S3: Results of the fitting of the EXAFS curves for sintered and nanoporous samples. The experimental data (k-range: 3-12 $Å^{-1}$, R-range: 1.0-2.8 Å) were fitted in R-space (k-weighting=2). For pristine s-LSTN and n-LSTN, the R-range was 1.0-3.0 Å, to include the Ni-M distance in the LSTN structure.

**Sample**	**Scattering path**	**Relative amount [%]**	**Distance [Å]**	**Debye-Waller factor [10^{-3} $Å^2$]**	**E_0 [eV]**	**R-factor [%]**
s-LSTN	Ni-O	100	2.02 ± 0.01	2.7 ± 0.9	-2.3 ± 1.2	0.86
	$Ni\text{-}M_{LSTN}$		2.94 ± 0.01	2.8 ± 0.5		
s-LSTN R700	Ni-O	87.0 ± 5.9	2.02 ± 0.01	1.2 ± 1.0	-1.7 ± 1.8	1.36
	Ni-Ni	13.0 ± 5.9	2.49 ± 0.01	6.2 ± 4.3		
s-LSTN R800	Ni-O	65.9 ± 3.3	2.01± 0.01	0.9 ± 0.7	-0.6 ± 1.3	0.44
	Ni-Ni	34.1 ± 3.3	2.51 ± 0.01	6.2 ± 0.9		
s-LSTN R900	Ni-O	54.8 ± 4.9	2.01 ± 0.01	1.4 ± 1.3	-1.1 ± 2.0	0.89
	Ni-Ni	45.2 ± 4.9	2.51 ± 0.01	6.3 ± 1.0		
n-LSTN	Ni-O	100	2.02 ± 0.01	3.6 ± 0.5	-1.6 ± 0.7	0.92
	$Ni\text{-}M_{LSTN}$		2.97 ± 0.01	2.6 ± 2.1		
n-LSTN R500	Ni-O	88.1 ± 7.2	2.02 ± 0.02	2.9 ± 1.4	-1.8 ± 2.6	2.30
	Ni-Ni	11.9 ± 7.2	2.49 ± 0.02	4.9 ± 5.1		
n-LSTN R700	Ni-O	60.5 ± 5.1	2.01 ± 0.01	2.1 ± 1.3	-1.0 ± 2.1	0.92
	Ni-Ni	39.5 ± 5.1	2.50 ± 0.01	5.8 ± 1.1		
n-LSTN R800	Ni-O	34.6 ± 3.7	2.01 ± 0.01	0.5 ± 1.2	-1.3 ± 1.2	0.35
	Ni-Ni	65.4 ± 3.7	2.49 ± 0.01	5.7 ± 0.5		
n-LSTN R900	Ni-O	21.3 ± 4.6	2.00 ± 0.02	1.7 ± 2.9	-1.5 ± 1.1	0.35
	Ni-Ni	78.7 ± 4.6	2.49 ± 0.01	5.4 ± 0.5		

Table S4: Atomic composition of the pristine materials

**Sample**	**La [% at.]**	**Sr [% at.]**	**Ti [% at.]**	**Ni [% at.]**
n-LSTN	50	30	94	6
s-LSTN	49	31	95	5

Table S5: Results of Arrhenius plots of the extent of metallic Ni (Ni^0_{XANES}) from XANES spectroscopy of n-LSTN and s-LSTN versus reduction temperature with reliability factors Pearson R, R^2 and adjusted R^2.

Sample	Activation Energy [kJ·mol^{-1}]	Pearson R	R^2 (COD)	Adj. R^2
s-LSTN	82 ± 32	-0.93014	0.86515	0.73031
n-LSTN	45 ± 3	-0.99434	0.9887	0.98306

Table S6: Summary of the reliability factors X^2 and R-factor from the linear combination fitting (LCF) from the XANES results.

Sample	R-factor (%)	X^2
s-LSTN R700	0.13299	0.03902
s-LSTN R800	0.13026	0.03579
s-LSTN R900	0.09336	0.02181
n-LSTN R500	0.02348	0.00663
n-LSTN R700	0.10002	0.02378
n-LSTN R800	0.02411	0.00724
n-LSTN R900	0.21855	0.04295
n-LSTN Red1	0.00742	0.00247
n-LSTN Red2	0.02768	0.00926
n-LSTN Red3	0.04036	0.01343
n-LSTN Ox1	0.01675	0.00584
n-LSTN Ox2	0.03563	0.01245

Table S7: Summary of the parameters for the lognormal fitting of the SAXS curves of the nanostructured exsolved materials: particle radius (R), variance (σ), number density times scattering contrast square of particles ($N\eta^2$), background (c_0) reliability factors of the fit (χ^2, R^2).

Sample	R [nm]	σ [nm]	$N\eta^2$	c_0	χ^2	R^2
n-LSTN R500	7.08	10.65	9.3e-4	4.02	1.39543	0.99963
n-LSTN R700	7.45	10.15	8E-4	3.45	1.48714	0.99962
n-LSTN R800	8.49	11.46	6.1E-4	3.44	0.78152	0.99963
n-LSTN R900	9.56	12.75	3.1E-4	3.57	2.12844	0.99898

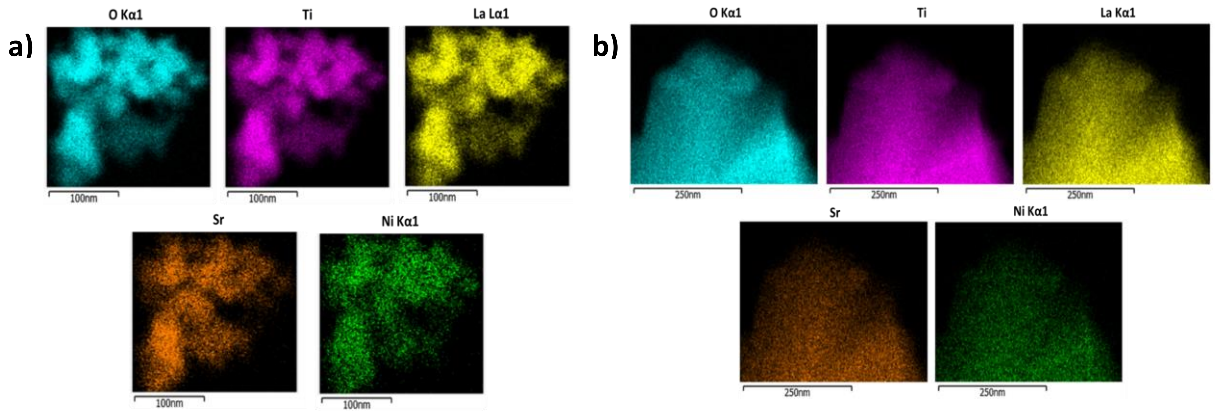

Figure S1: TEM-EDX mapping of the elements in the pristine materials n-LSTN (a) and s-LSTN (b).

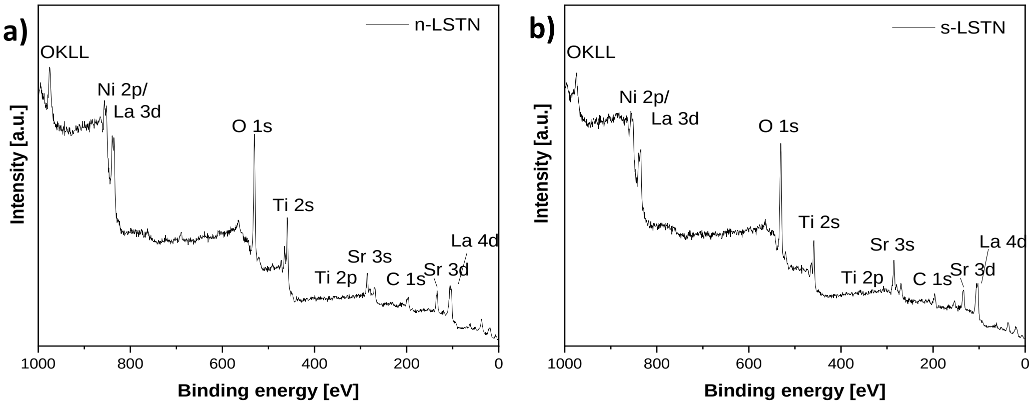

Figure S2: Survey XPS of the pristine materials n-LSTN (a) and s-LSTN (b).

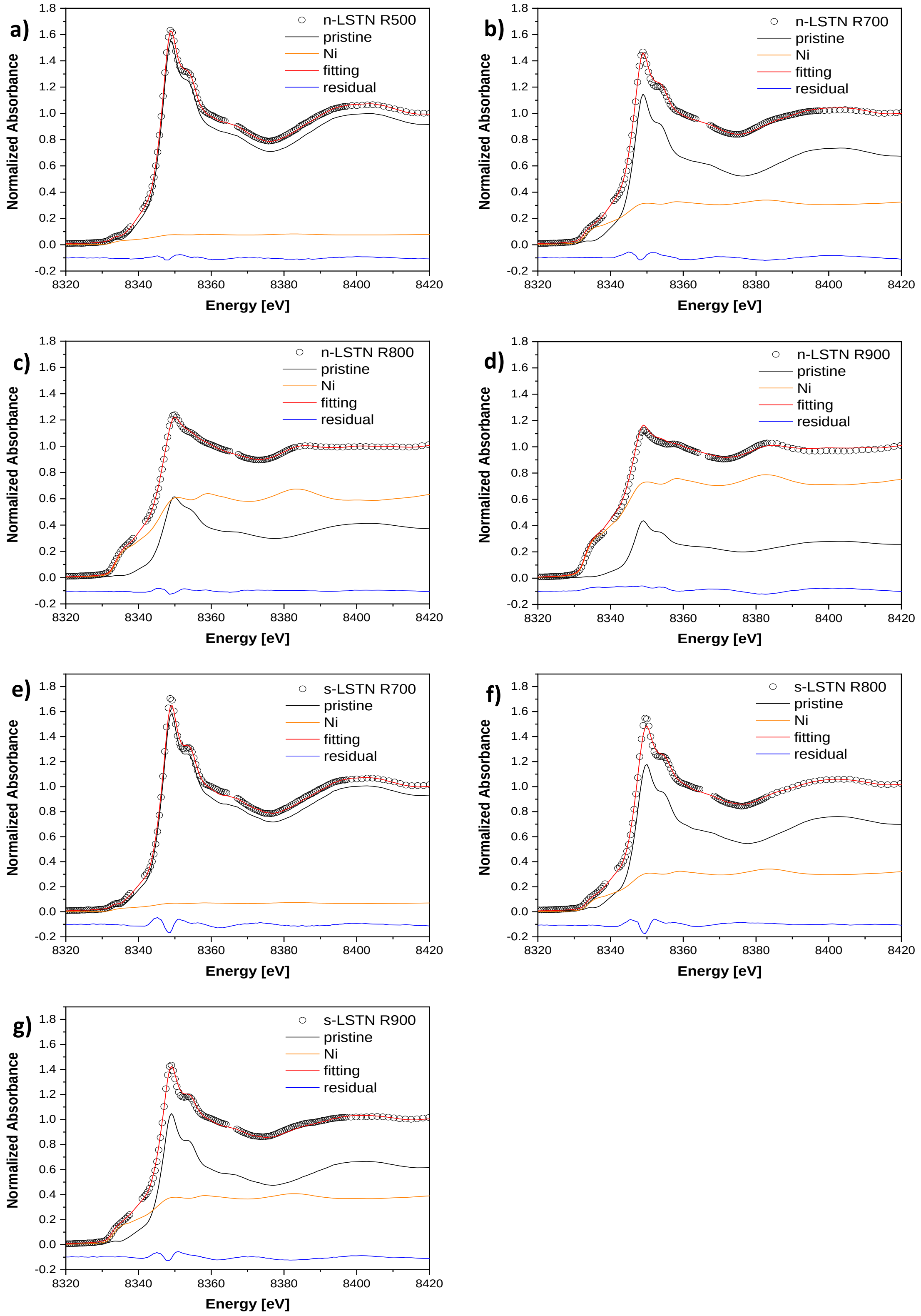

Figure S3: Ni K-edge ANES spectra of n-LSTN (a-d) and s-LSTN (e-g) materials after exsolution with their linear combination fittings containing the weighted components of the Ni in the pristine matrix (black line), metallic Ni (orange line), the linear combination fitting (red line) and difference plots (residual, blue line) obtained from $A^{exp} - A^{fit}$, with A the normalized absorbance for the exsolved materials.

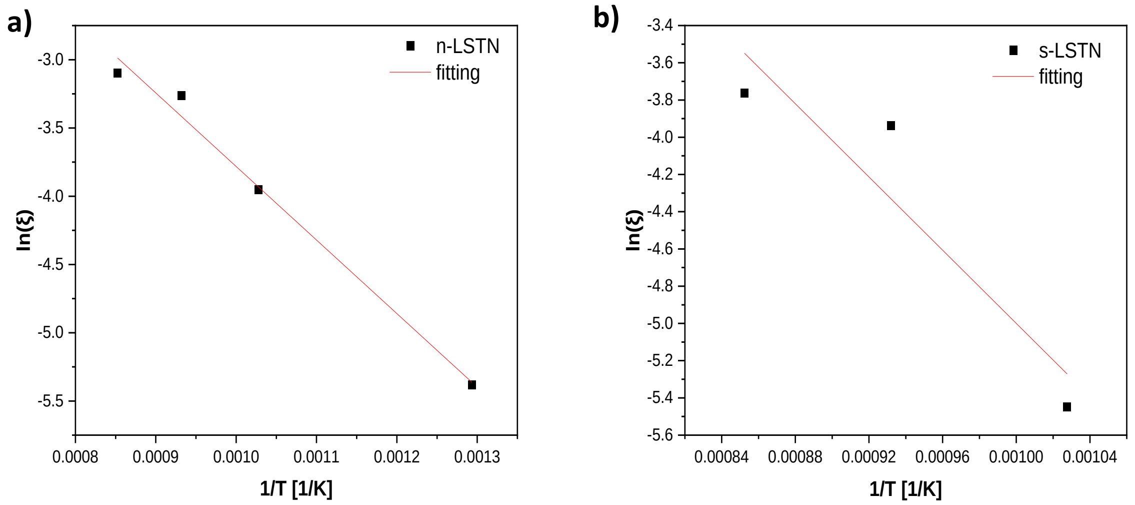

Figure S4: Arrhenius plots of the extent of metallic Ni (ξ) from XANES spectroscopy of n-LSTN (a) and s-LSTN (b).

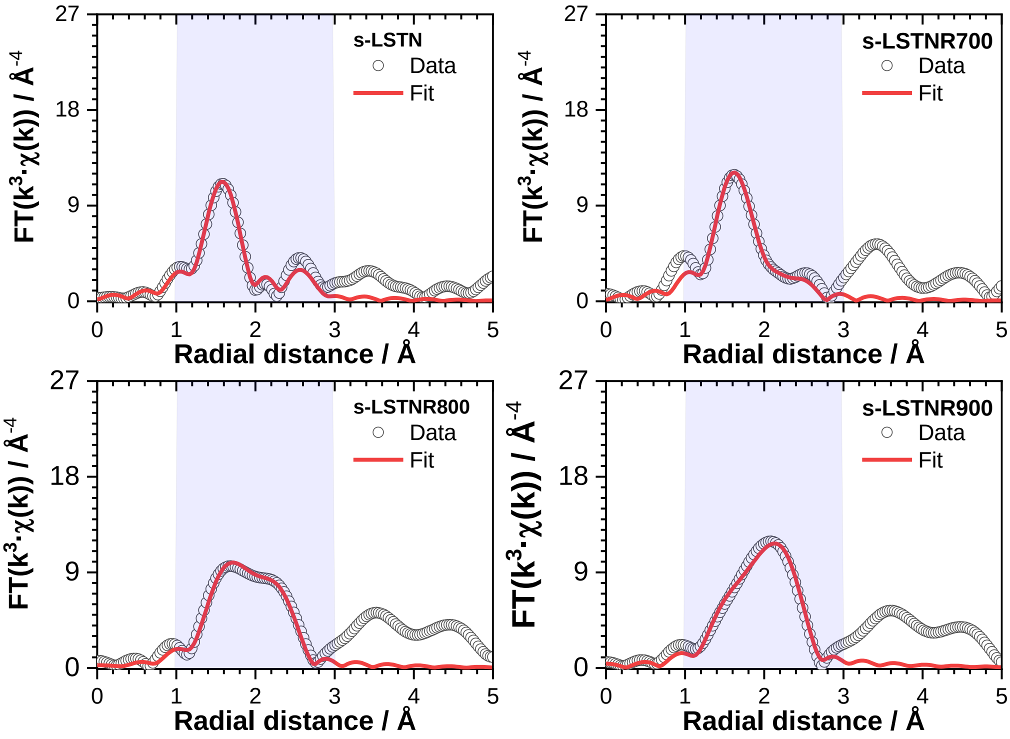

Figure S5: Fourier transform of the k^3-weighted EXAFS curves (empty dots) and fit model (red line) for the sintered samples. The light blue shadow indicates the fitted region.

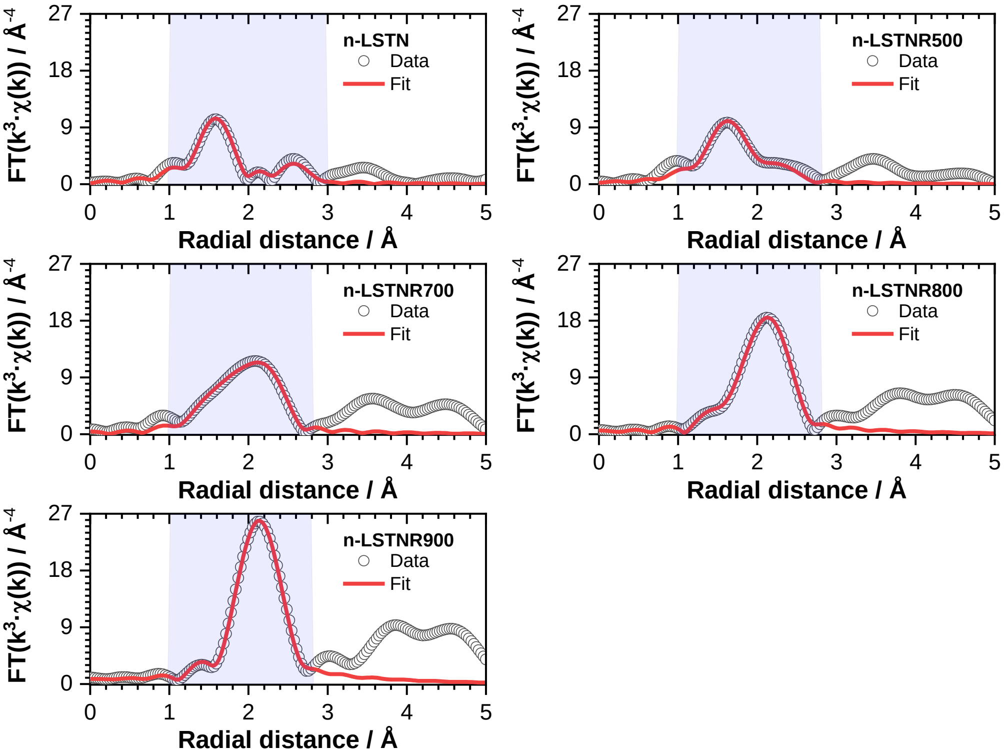

Figure S6: Fourier transform of the k^3-weighted EXAFS curves (empty dots) and fit model (red line) for the nanoporous samples. The light blue shadow indicates the fitted region.

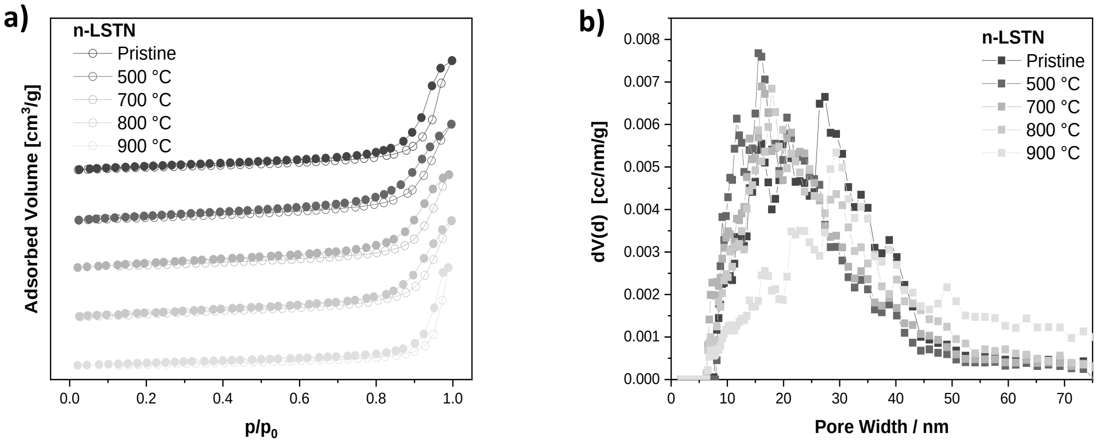

Figure S7: Nitrogen physisorption isotherms (a) of n-LSTN before and after exsolution with respective pore size distribution plots (b). Each isotherm was upshifted upward by 50 $cm^3 \cdot g^{-1}$ for clarity except for the data referring to n-LSTNR900.

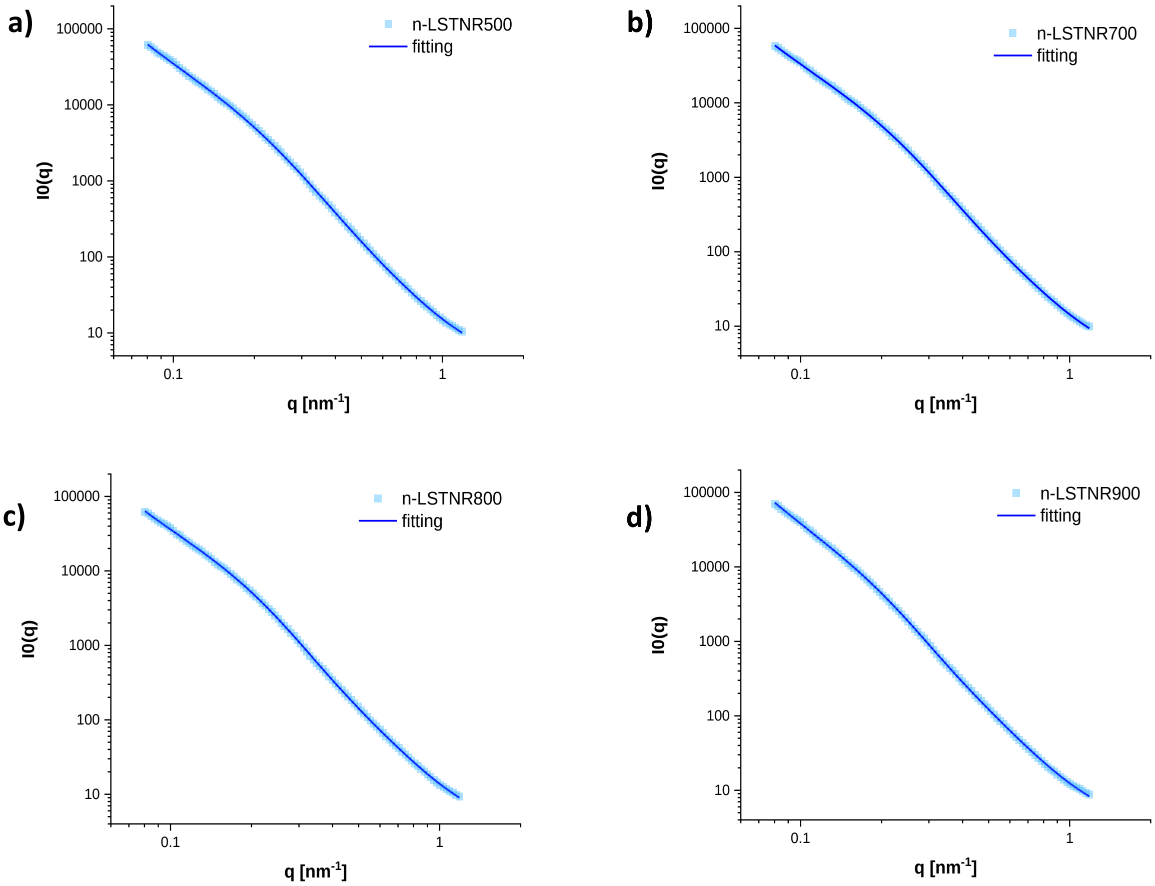

Figure S8: SAXS curves of n-LSTN reduced at 500 °C (a), 700 °C (b), 800 °C (c), 900 °C (d)

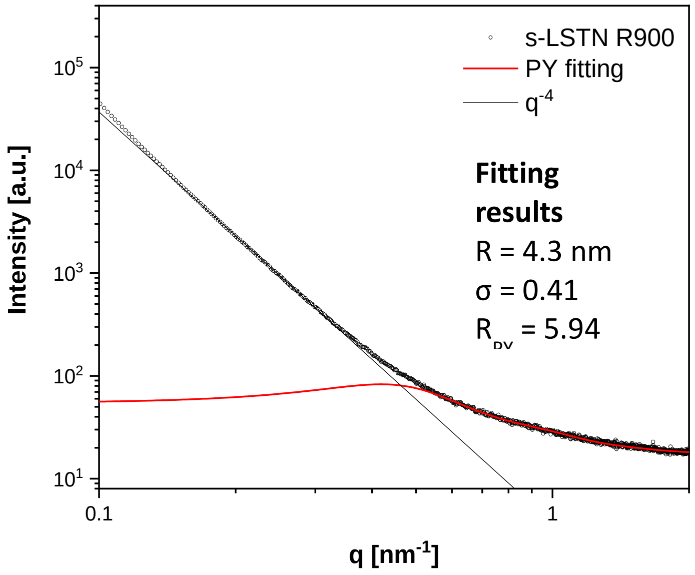

respectively fitted with a lognormal distribution function.

Figure S9: SAXS curve of s-LSTN exsolved at 900 °C with Percus-Yevick (PY) fitting results: particle radius (R), size polydispersity (σ), particle-to-particle distance (R_{PY}) and particle density (η).

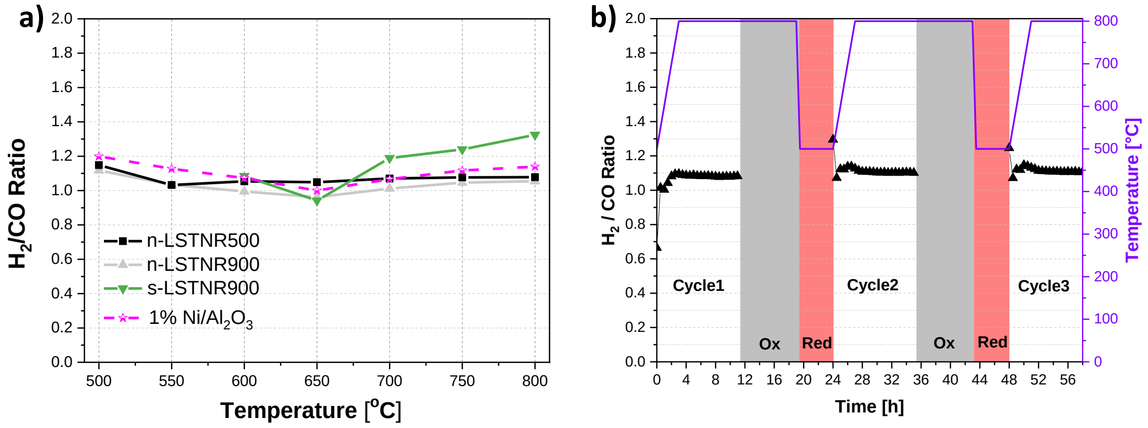

Figure S10: Plots of the produced H_2/CO molar ratio of different Ni-based catalysts during time on stream experiment (a) and of the n-LSTNR500 during 3 catalytic cycles (b).

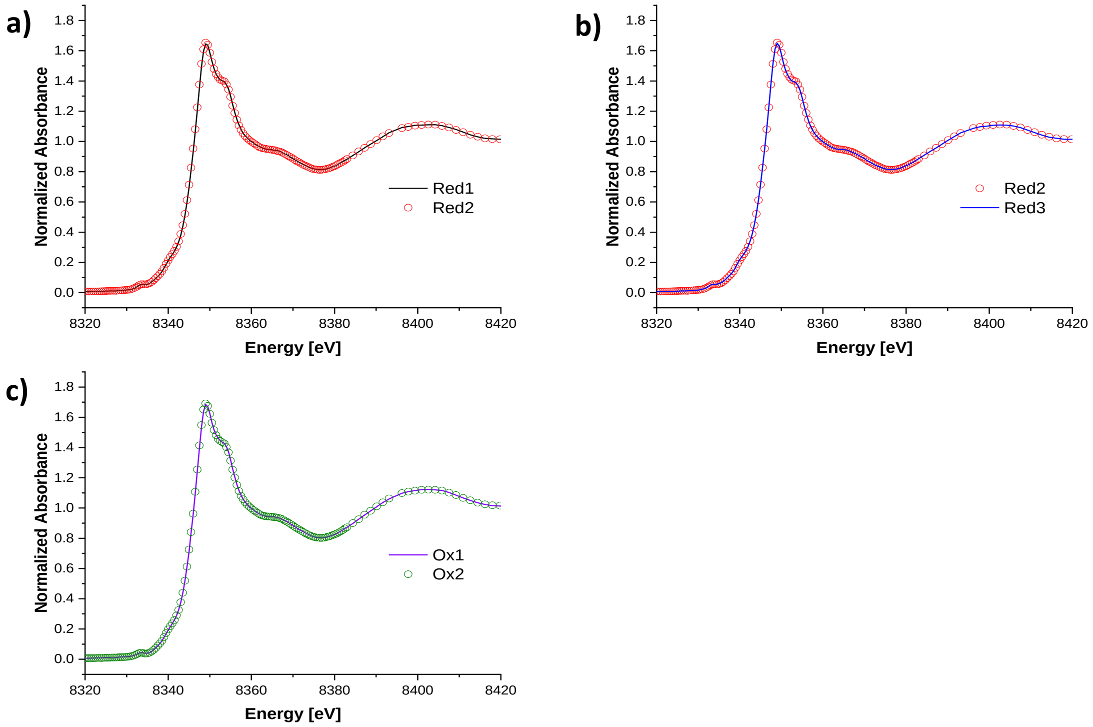

Figure S11: XANES spectra at Ni K-edge of n-LSTNR500 after first and second reduction (a), after second and third reduction (b), after first and second oxidation (c).

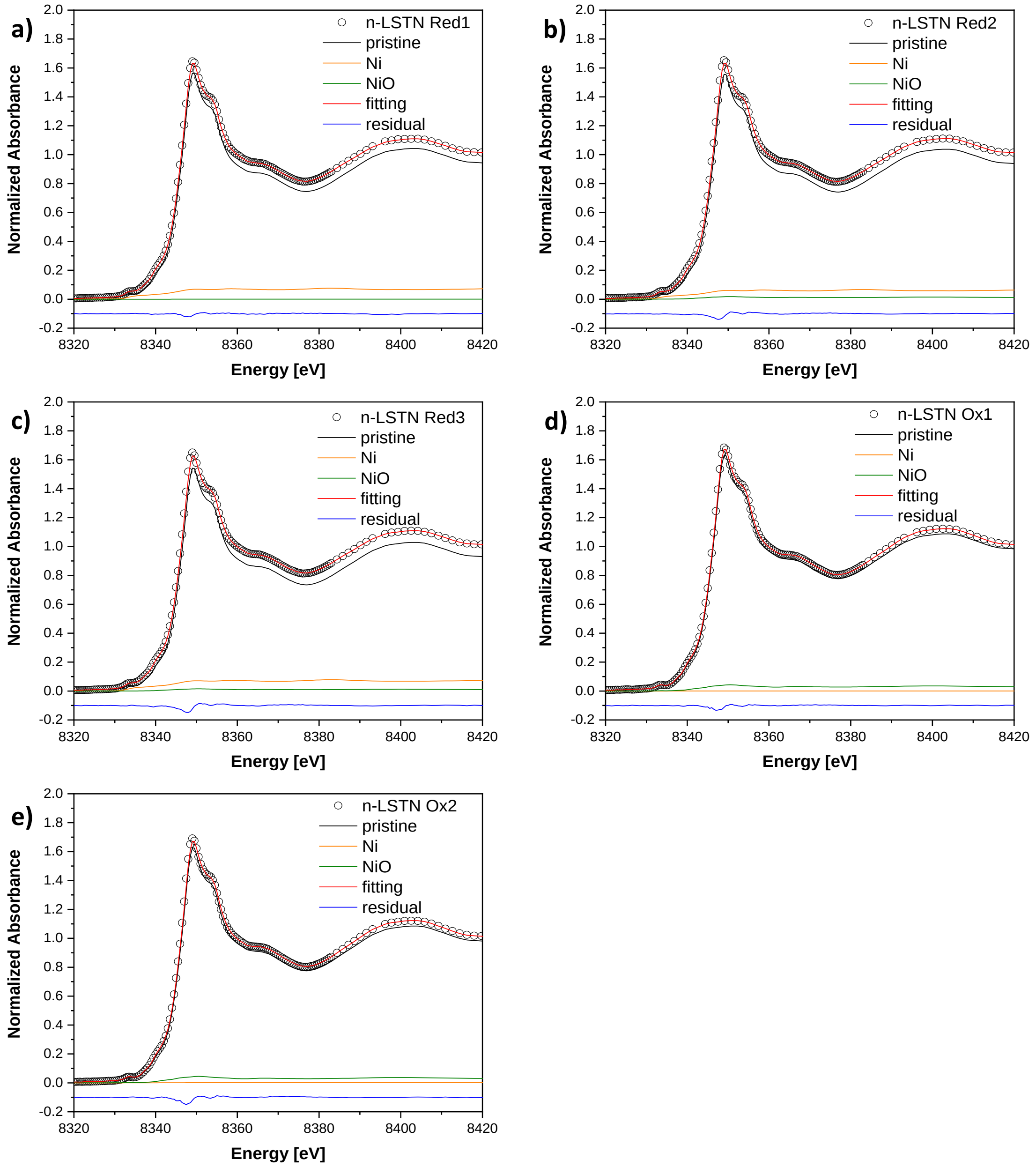

Figure S12: XANES spectra at the Ni K-edge of n-LSTNR500 after reductions (a-c) and oxidations (d,e) with their linear combination fittings containing the weighted components of the Ni in the pristine matrix (black line), metallic Ni (orange line), the linear combination fitting (red line) and difference plots (residual, blue line) obtained from $A^{exp} - A^{fit}$, with A the normalized absorbance for the exsolved materials
